# miR-126 promotes M1 to M2 macrophage phenotype switching *via* VEGFA and KLF4

**DOI:** 10.7717/peerj.15180

**Published:** 2023-03-31

**Authors:** Xinyang Shou, Yimin Wang, Qingyu Jiang, Jun Chen, Qiang Liu

**Affiliations:** 1 Zhejiang Chinese Medical University, Hangzhou, China; 2 The First Affiliated Hospital of Zhejiang Chinese Medical University, Hangzhou, China; 3 The Third Affiliated Hospital of Zhejiang Chinese Medical University, Hangzhou, China

**Keywords:** miR-126, Macrophage phenotype, ox-LDL, KLF4

## Abstract

**Background:**

Macrophage polarization and microRNA play crucial roles in the development of atherosclerosis (AS). The M1 macrophage phenotype contributes to the formation of plaques, while the M2 macrophage phenotype resolves inflammation and promotes tissue repair. MiR-126 has been found to play a role in regulating macrophage polarization in the context of AS. However, the exact mechanism of miR-126 requires further research.

**Methods:**

The foam cell model was established by stimulating THP-1 with oxidized low-density lipoprotein (ox-LDL). We transfected foam cells with miR-126 mimic and its negative control. The transfection of miR-126 was implemented by riboFECT CP transfection kit. The levels of miR-126 and M1/M2 associated genes in foam cells were quantified using reverse transcription-quantitative PCR (RT-qPCR). Additionally, the expressions of CD86^+^ and CD206^+^ cells in foam cells were determined by flow cytometry. Western blotting and RT-qPCR were used to determine the protein and mRNA levels of the vascular endothelial growth factor A (VEGFA) and the transcriptional regulator Krüppel-like factor 4 (KLF4), respectively. Additionally, we detected endothelial cell migration after co-culturing endothelial cells and macrophages. MG-132 was used to indirectly activate the expression of VEGFA, and the expression of KLF4 was also evaluated.

**Results:**

The activation of apoptosis and production of foam cells were boosted by the addition of ox-LDL. We transfected foam cells with miR-126 mimic and its negative control and observed that miR-126 greatly suppressed foam cell development and inhibited phagocytosis. Moreover, it caused pro-inflammatory M1 macrophages to switch to the anti-inflammatory M2 phenotype. This was reflected by the increase in anti-inflammatory gene expression and the decrease in pro-inflammatory gene expression. Additionally, miR-126 dramatically decreased the expressions of VEGFA and KLF4. The protein-protein interaction network analysis showed a significantly high correlation between miR-126, VEGFA, and KLF4. MiR-126 may also promote EC migration by activating macrophage PPAR γ expression and effectively suppressing macrophage inflammation. MG-132 indirectly activated the expression of VEGFA, and the expression of KLF4 also significantly increased, which indicates a direct or indirect relationship between VEGFA and KLF4.

**Conclusion:**

Our study shows that miR-126 can reverse ox-LDL-mediated phagocytosis and apoptosis in macrophages. Consequently, the potential role of miR-126 was manifested in regulating macrophage function and promoting vascular endothelial migration.

## Introduction

Atherosclerosis (AS) is now recognized as a chronic inflammatory disease and considered to be the primary pathophysiologic mechanism driving coronary artery disease (CAD) (Song et al., 2020; Zhou et al., 2019). AS is also the result of plaque buildup in the arteries, primarily caused by hyperlipidemia (Filipek et al., 2020). Hyperlipidemia, a component of metabolic syndrome, results in an abnormal rise in very-low-density lipoprotein cholesterol, low-density lipoprotein cholesterol, total cholesterol (TC), and triglyceride (TG) levels, which contribute to the development of AS (Panahi et al., 2018). The plasma level of oxidized low-density lipoprotein (ox-LDL) is a powerful predictor of CAD and a critical factor in the development of AS (Wolf & Ley, 2019).

Inflammatory activities such as endothelial cell (EC) activation and recruitment, monocyte infiltration, macrophage polarization, and foam cell production are triggered by elevated lipids mentioned above. All these factors eventually contribute to the production of atherosclerotic plaques. Through the phagocytosis of ox-LDL in AS, monocytes accumulate under vascular ECs and differentiate into macrophages (Qin, 2012). The macrophage phenotype polarization can affect the stability of atherosclerotic plaques in human arteries (Filipek et al., 2020). Macrophages are traditionally categorized as M1, M2, Mox, and Mhem and defined according to a small number of markers (Colin, Chinetti-Gbaguidi & Staels, 2014). Single-Cell RNA-Seq has already been applied in murine atherosclerosis to classify aortic macrophages into inflammatory macrophages, resident-like (Res-like) macrophages, and TREM2hi (triggered receptor expressed on myeloid cells 2) macrophages (Cochain et al., 2018). Although there are many ways to classify macrophages, the most conventional classification is M1 and M2. In M1, a high level of proinflammatory cytokines with immunostimulatory activity is produced (Xiao et al., 2018). M2 is believed to modulate M1 responses by stimulating inflammation resolution and inducing tissue healing (Porcheray et al., 2005). Thus, inflammation subsides in an M2 macrophage-enriched environment and tissue remodeling occurs (Porcheray et al., 2005). Atherogenesis is associated with unstable plaque production due to the presence of M1 macrophages, whereas M2 macrophages are associated with stable plaque formation (Wu et al., 2020). Dyslipidemia is a major stimulatory factor for pro-inflammatory M1 responses and promotes the development of atherosclerotic plaques (Moore, Sheedy & Fisher, 2013). CD206 is a membrane-bound protein primarily expressed in M2 macrophages and dendritic cells, while CD86 is mainly expressed in M1 macrophages (Xiao et al., 2018; Xu et al., 2018).

MicroRNAs adversely influence gene expression through translational inhibition or the destruction of target messenger RNAs in CAD (Saliminejad et al., 2019). MicroRNA-126 (miR-126) is primarily expressed in ECs, which is essential for the preservation of endothelial proliferation and the promotion of endothelial growth and repair (Chistiakov, Orekhov & Bobryshev, 2016; Fish et al., 2008). Meanwhile, miR-126 is also expressed in platelets and red blood cells (McCall et al., 2017). The serum miR-126 was found to be significantly downregulated in AS patients (Jiang et al., 2014). Both distinct mature miR-126-5p and miR-126-3p arise from the same pre-miR. miR-126-5p is increased by laminar shear stress and induces EC proliferation at preferred sites with ongoing inflammation and endothelial apoptosis (Boon & Dimmeler, 2014). Researchers discovered that endothelial recovery after denudation was impaired in miR126^−^/^−^ mice because the lack of miR-126-5p, but not miR-126-3p, reduced EC proliferation (Schober et al., 2014). Furthermore, miR-126 regulated macrophage recruitment and induced a reduction in the level of pro-inflammatory cytokines (Wu et al., 2022). Therefore, we hypothesized that miR-126 influences macrophage polarization, from M1 to M2, and may have a beneficial effect on AS.

Vascular endothelial growth factor (VEGF) is a primary inducer of angiogenesis. High expression of miR-126 inhibited VEGF secretion (Ge et al., 2015). The loss of miR-126 increases vascular permeability and leakage due to the upregulation of expression of VEGF inhibitors (Fish et al., 2008; Wang et al., 2008). In other words, VEGF expression can be inhibited by miR-126 in mature vessels (Chistiakov, Orekhov & Bobryshev, 2016). Cell proliferation and differentiation are two of the many functions that Krüppel-like factor 4 (KLF4) displays in monocytes, wherein this DNA-binding transcriptional regulator is highly expressed (Alder et al., 2008; El-Karim et al., 2013). KLF4 exerts an anti-atherogenic effect by aiding macrophage polarization in adipose tissue (Liao et al., 2011). KLF4-deficient macrophages exhibit increased expression of proinflammatory genes, suggesting that KLF4 promotes M2 polarization (Liao et al., 2011). However, the mechanism of action of the VEGFA/KLF4 axis needs to be investigated.

In this study, miR-126 was transfected into macrophages to study macrophage polarization. We examined the phagocytosis efficiency, apoptosis rate, and expression of macrophage membrane proteins. The mRNA expression of *TNFA*, *CCL2*, *CCL18*, *CD206*, and *PPAR γ*, and the protein expressions of the VEGFA and KLF4, were examined. We also co-cultured ECs and macrophages to observe the proliferation of ECs. We further clarified the relationship between VEGFA and KLF4 by indirectly stimulating VEGFA.

## Materials and Methods

### Cell culture

The THP-1 monocyte (SCSP-567) was an established cell line directly purchased from the Chinese Academy of Sciences cell bank (Shanghai, China) on February 10^th^, 2021. Human umbilical vein endothelial cells (HUVEC, PUMC-HUVEC-T1) were obtained from the Cell Resource Center, Peking Union Medical College (the headquarters of National Science & Technology Infrastructure--National BioMedical Cell-Line Resource, NSTI-BMCR) on October 15^th^, 2022. Cells were cultured in 10% (v/v) fetal bovine serum (FBS; Sijiqing Bioengineering Material Co., Ltd, Zhejiang, China) and RPMI 1640 medium (Gibco, Shanghai, China) in a humidified incubator with 5% CO_2_ at 37 °C. HUVEC was cultured in high-glucose DMEM (Gibco, Shanghai, China) supplemented with 10% FBS at 37 °C in 5% CO_2_. THP-1 cells were seeded at a density of 5 × 10^5^ cells/well in a 6-well plate. Within 48 h of incubation with 100 ng/mL PMA (Sigma-Aldrich, St. Louis, MO, USA) (Wang et al., 2019; Zhong et al., 2020), THP-1 cells differentiated into adherent THP-1-Mφ. Foam cells were grown with adherent THP-1-Mφ and 50 μg/mL ox-LDL (Yiyuan Biotech. Co. Ltd, Guangdong, China). The solution of 1 uM MG-132 (Z-Leu-Leu-Leu-al, MedChamExpress, Monmouth Junction, NJ, USA) was added to replace ox-LDL solution after 24 h treatment and cultured with foam cells for 4 h.

### Oil red O staining assays

After being fixed in 4% paraformaldehyde at 23 °C for 10 min, the cells were rinsed thrice in 1% PBS. The plate was rinsed with washing solution for 30 s, oil red O solution (Beyotime, Shanghai, China) was added, and the plate was incubated at 23 °C for 30 min. The oil red O solution was washed away for 20 s using the washing solution. Cells were then immediately washed four times with 1 × PBS. Images were acquired under a microscope (Olympus, Tokyo, Japan) and analyzed using the Image J software (NIH, Bethesda, MD, USA).

### Transfection

MicroRNA transfection was performed using riboFECT CP transfection kit (RiboBio Co., LTD, Guangzhou, China). Briefly, 10 µL of 20 µM riboFECT CP mimic and NC were diluted with 120 µL of 1 × riboFECT CP transfection buffer and 12 µL of riboFECT CP transfection reagent was added. Then, it was gently vortexed and incubated at 23 °C for 15 min. Subsequently, the mixture was diluted to 2 mL with 10% FBS RPMI 1640 media, and added to the adhered cells. Adherent cells were grown in 10% FBS RPMI 1640 media containing 100 nM mimic or 100 nM negative control of miR-126-5p (Ribo Biotech Co., Ltd, Guangdong, China) for 24 h with 50 μg/mL ox-LDL (Yiyuan Biotech. Co. Ltd, Guangdong, China). Next, a blank group was set as the control, and a group with only ox-LDL was treated as the model group.

### Transwell and crystal violet assays

THP-1 cells differentiation and transfection were performed as mentioned above. HUVEC was seeded in chambers (#14112, PET Polyester, 0.4 μm, 24 mm, Labselect, Anhui, China) and transduced the next day for 24 h. The procedure is detailed in Supplemental 1. HUVEC was fixed for 15 min with 4% paraformaldehyde at 23 °C. Subsequently, chambers were washed by submersion in a water bath, followed by permeation using anhydrous methanol for 20 min at 23 °C. For crystal violet staining, chambers were aspirated and 0.5% crystal violet (Beyotime, Shanghai, China) was added for the cells from the bottom of the chamber. After 10 min at 23 °C, crystal violet was removed, and the chambers were thoroughly washed thrice using PBS. Images were acquired under a microscope (Olympus, Tokyo, Japan) and analyzed using the Image J software (NIH, Bethesda, MD, USA).

### Flow cytometry

FITC Annexin V Apoptosis Detection Kit I (BD Pharmingen, San Diego, CA, USA), APCanti-human CD86 antibody (Biolegend, Beijing, China), and FITC anti-human CD206 antibody (Biolegend, Beijing, China) were applied following the manufacturer’s instructions. We obtained adherent macrophages using the Trypsin-Digestion Method. After collecting the original 10% FBS culture medium, cells were washed four times with 1 × PBS, followed by washing twice with 0.25% EDTA-free-Trypsin (Beyotime, Shanghai, China). Then, 0.5 mL 0.25% EDTA-free-Trypsin was added to the cells to digest for 5 min and digestion was stopped using original 10% FBS culture medium.

A concentration of 1 × 10^6^ cells per milliliter of the binding solution was utilized to collect and wash the cells thrice before resuscitation in 1 × binding buffer. Cells were treated with 5 µL of FITC antibody, propidium iodide (PI), CD86 antibody, and CD206 antibody at 23 °C for 25 min avoiding light exposure. Finally, 400 µL of 1 × binding buffer was added to each tube, followed by analysis with a flow cytometer (FACS Calibur, BD Biosciences, Franklin Lakes, NJ, USA) within 1 h. For apoptosis, Quadrants Q1 and Q2 were FITC^+^ (early and late apoptotic cells), Q3 FITC^−^/PI^+^ (dead cells), and Q4 FITC^−^/PI^−^ (intact cells). For macrophage phenotype polarization, we firstly gated out the dead cells which from Quadrants Q12 and Q10 were CD86^−^CD206^−^ and CD86^+^CD206^+^ (meaningless in our research), Q9 was CD86^−^CD206^+^ (M2 macrophages), and Q11 was CD86^+^CD206^−^ (M1 macrophages). The FlowJo software was used to collect and analyze the data (Tree Star Inc., Ashland, OR, USA).

### TC and TG assays

TC and TG were assayed with Cholesterol Assay and TG Quantification Kits (Jiancheng Bioengineering Institute, Jiangsu, China). Samples were processed following the manufacturer’s instructions. Standards were set at 2.26 mmol/L (TG) and 5.17 mmol/L (TC). The final concentrations of TGs and TCs were normalized for protein content.

### Measurement of gene expression by real-time RT-PCR

Total RNA was extracted by Trizol following the manufacturer’s instructions, precipitated with isopropanol, and washed with ethanol. It was then converted to cDNA using Evo M-MLV RT Premix for qPCR (Accurate Biotechnology, Hunan, China) and analyzed by RT-PCR with a Roche LightCycler 96 system (Roche, Basel, Switzerland) using the SYBR Green Premix Pro Taq HS qPCR Kit (Accurate Biology). The following primers were used in this experiment: *TNFA*, *CCL2* (for M1), *CCL18*, *PPAR γ*, *CD206* (for M2), *KLF4*, *VEGF*, and human *ACTB* (Sangon Biotech, Shanghai, China, B661102-0001). All the primer sequences used in this investigation are listed in Table 1. The expression of each gene was assessed by comparing it to the expression of β-actin using the 2^−ΔΔCt^ method.

**Table 1:** Primers for RT-qPCR.

Gene	Forward (5′–3′)	Reverse (5′–3′)	Amplification efficiency %
*CCL2*	GAAAGTCTCTGCCGCCCTTCTG	GCCTCTGCACTGAGATCTTCCTATTG	91.27858
*CCL18*	TCGTCTATACCTCCTGGCAGATTCC	CCACTTCTTATTGGGGTCAGCACAG	101.6721
*CD206*	AACGGACTGGGTTGCTATCA	CCCGATCCCTTGTAGAGCAT	92.26729
*KLF4*	ACCAGGCACTACCGTAAACACA	GGTCCGACCTGGAAAATGCT	104.9477
*PPAR γ*	GGGATCAGCTCCGTGGATCT	TGCACTTTGGTACTCTTGAAGTT	95.55322
*TNFA*	GAGGCCAAGCCCTGGTATG	CGGGCCGATTGATCTCAGC	109.34
*VEGFA*	ATTGAAATCAGCCAGCACGC	AGGAACCACAGTGCCAGATCC	90.59524

### Extraction of total proteins and western blotting

Total protein was extracted by centrifugation at 12,000 × *g* for 10 min at 4 °C, by using 200 μL cold RIPA lysis solution with phosphatase and protease inhibitors. The Enhanced BCA Protein Assay Kit (Beyotime, Shanghai, China) was used to evaluate protein concentrations in the supernatant based on the standard quantity of protein (0.3 µg/mL). After electrophoresis, a 10% SDS-polyacrylamide gel was used to transfer the proteins to polyvinylidene fluoride membranes in equal proportions. The gel was incubated overnight at 4 °C after blocking non-specific sites on the membranes. Western blotting was performed using the antibodies listed below: anti-β-Actin (13E5) rabbit mAb (1:1,000 dilution, #4970; CST, MA, USA); anti-KLF4 (D1F2) rabbit mAb (1:1,000 dilution, #12173; CST, Danvers, MA, USA); anti-VEGFA rabbit mAb (1:1,000 dilution, ab214424, Abcam, Waltham, MA, USA); anti-c-Jun (phospho S63) antibody (1:1,000 dilution, ab32385; Abcam, Waltham, MA, USA); c-Jun (60A8) Rabbit mAb (1:1,000 dilution, #9165, CST, Danvers, MA, USA); and anti-c-Fos antibody (1:1,000 dilution, ab134122; Abcam, MA, USA). The membranes were incubated for an hour at 23 °C with goat anti-rabbit HRP secondary antibody (1:5,000, ZB-2301; Zhongshanjinqiao, China). To detect protein bands, the Odyssey near-infrared two-color laser imaging system (LI-COR Biosciences, Lincoln, Nebraska) was employed. The band density of each sample was standardized to β-actin. Image J (NIH) was used to quantify the bands.

### PPI network and miRNA-target co-expression

The protein-protein interaction (PPI) network of EGFL7, VEGFA, and KLF4 was generated with STRING (https://cn.string-db.org). The line color of the network edges indicates the type of interaction. The minimum required interaction score was low confidence (0.150). We discovered a correlation between miR-126-5p and VEGFA in the starBase database (http://starbase.sysu.edu.cn/) Pan-Cancer (miRNA-target co-expression field).

### Co-culture

THP-1 cells were seeded at a density of 5 × 10^5^ cells/well in a 6-well plate. Within 48 h of incubation with 100 ng/mL PMA, THP-1 cells differentiated into adherent THP-1-Mφ. Adherent cells were grown in 10% FBS RPMI 1640 media containing 100 nM mimic or 100 nM negative control mimic of miR-126-5p for 24 h with 50 μg/mL ox-LDL. Next, a blank group was set as the control, and a group with only ox-LDL was treated as the model group.

HUVEC was cultured in high-glucose DMEM without 10% FBS at a density of 3 × 10^5^ cells/well in a chamber (#14112, LABSELECT, Anhui, China) for 24 h before putting the chambers in the 6-well plate. Refer to Supplemental 1 for more details.

### Statistical analysis

All data are expressed as mean ± SEM unless otherwise noted. Six sets of transfection samples were prepared, and two-way analysis of variance was used to establish statistical significance. One-way analysis of variance was used to assess the numerous remaining samples. The GraphPad Prism 9 software was used to conduct all statistical analyses (GraphPad, San Diego, CA, USA). All experiments were performed independently in triplicate.

## Results

### Ox-LDL induces foam cell formation and apoptosis in macrophages

**Figure 1:**
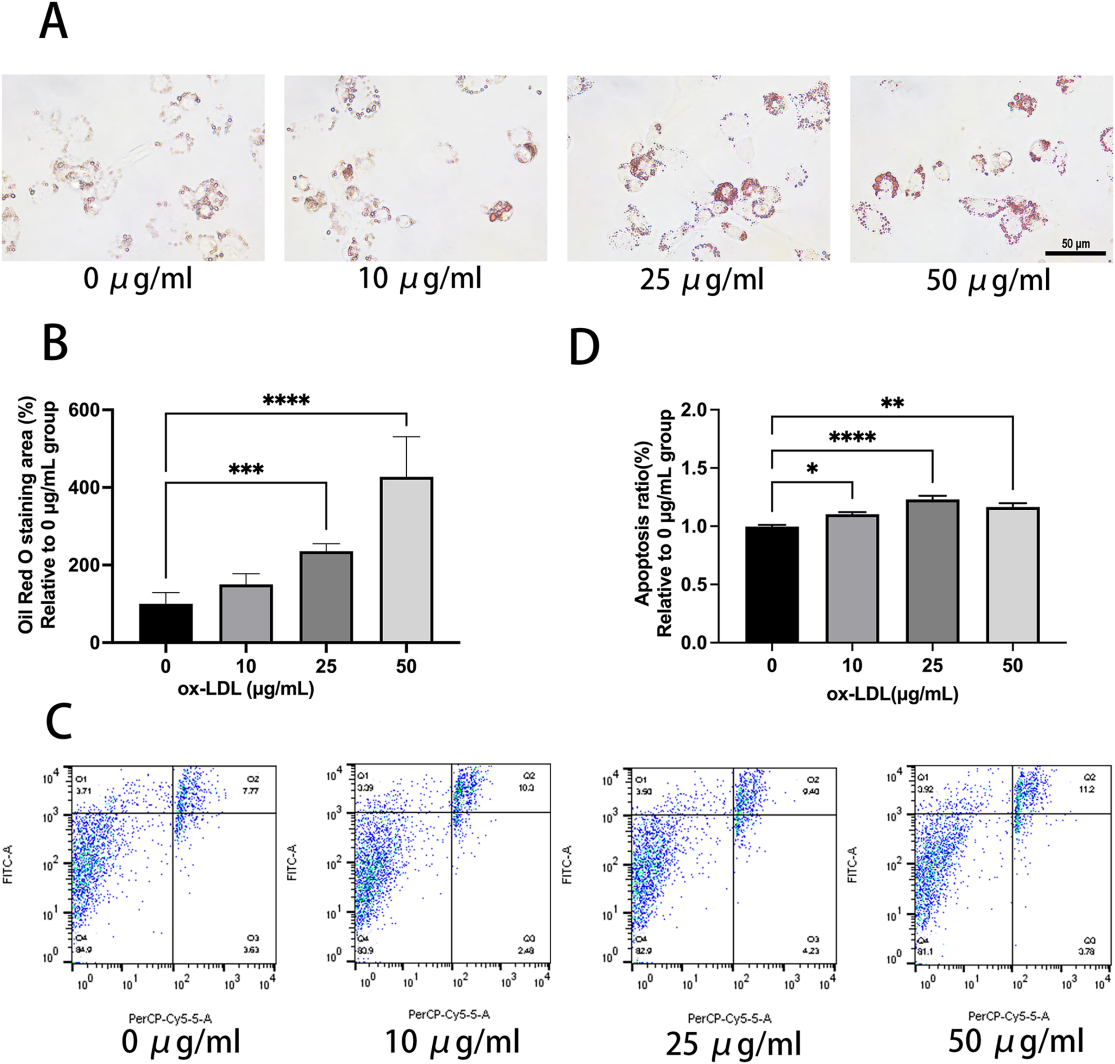
50 μg/mL Ox-LDL induced foam cell formation and triggers apoptosis. (A) Oil red staining area was measured in macrophages treated for 24 h with various concentrations of ox-LDL. Scale bar: 50 µm. (B) Quantification of lipid staining area from (A). Four asterisks (****) denote *P* value < 0.0001, three asterisks (***) denote *P* value < 0.001 by one-way ANOVA analysis (*n* = 4, >1,000 cells per trial). (C) Apoptosis rate of macrophages with various concentrations of ox-LDL for 24 h was calculated. (D) Quantification of dead cell ratio from (C). Four asterisks (****) denote *P* value < 0.0001, two asterisks (**) denote *P* value < 0.01, and one asterisk (*) denotes *P* value < 0.05 by one-way ANOVA analysis (*n* = 4, >1,000 cells per trial).

**Figure 2:**
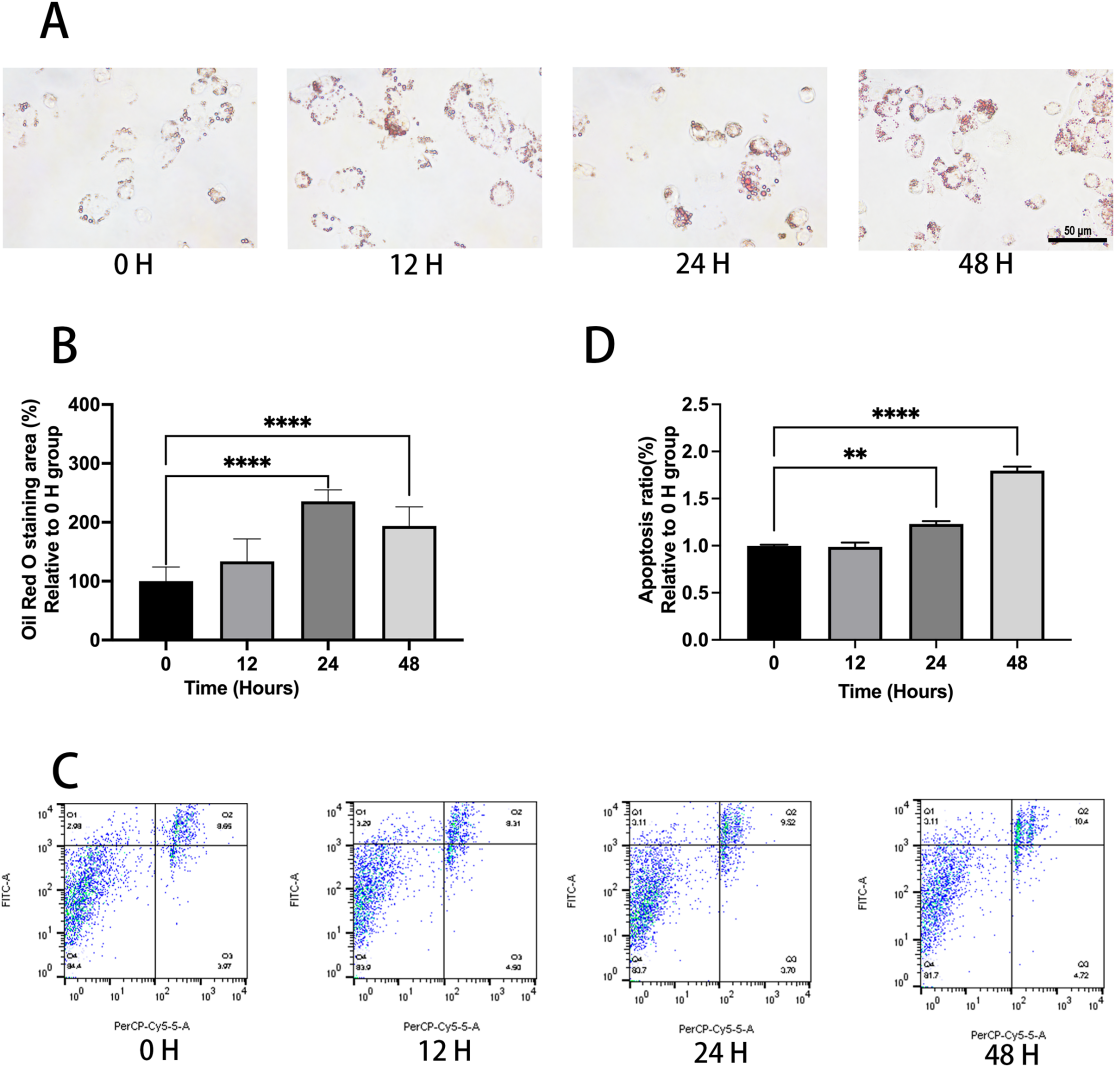
Ox-LDL induced foam cell formation and triggers apoptosis in 24 h. (A) Macrophages were treated with 50 μg/mL ox-LDL at different time intervals and stained with Oil red O. Scale bar: 50 µm. (B) The area of Oil Red O was evaluated from (A). Four asterisks (****) denote *P* value < 0.0001 by one-way ANOVA analysis (*n* = 4, >1,000 cells per trial). (C) Macrophages were treated with 50 μg/mL ox-LDL at different time intervals and the rate of apoptosis was measured. (D) Quantification of dead cell ratio from (C). Four asterisks (****) denote *P* value < 0.0001, and two asterisks (**) denote *P* value < 0.01 by one-way ANOVA analysis (*n* = 4, >1,000 cells per trial).

Ox-LDL was added to the macrophages at doses of 0, 10, 25, and 50 µg/mL for 24 h. Foam cell area was dependent on the ox-LDL dose (Figs. 1A and 1B). The apoptosis rates of the 10 and 25 µg/mL groups were 1.1, and 1.23 times higher than those of the 0 µg/mL group, respectively (Figs. 1C and 1D). However, the 50 µg/mL group apoptosis rate was 1.16, times higher than the 0 µg/mL group, which had no statistical difference when compared to the 25 µg/mL group. Therefore, the concentration of 50 µg/mL ox-LDL was chosen to treat macrophages for varied time periods (Fig. 2). Macrophages treated for 24 h significantly triggered foam cell formation when compared with the 0 h, and 12 h group (Figs. 2A and 2B). However, the foam cell area of 48 h group was not larger than 24 h group and has no statistical difference from the 24 h group. The apoptosis ratio of macrophages in the 48 h group was much higher than the other groups (Figs. 2C and 2D). Thus, 50 µg/mL ox-LDL and 24 h were confirmed to be the conditions of the transfection experiment.

### MiR-126 inhibits foam cell formation and regulates lipid metabolism

**Figure 3:**
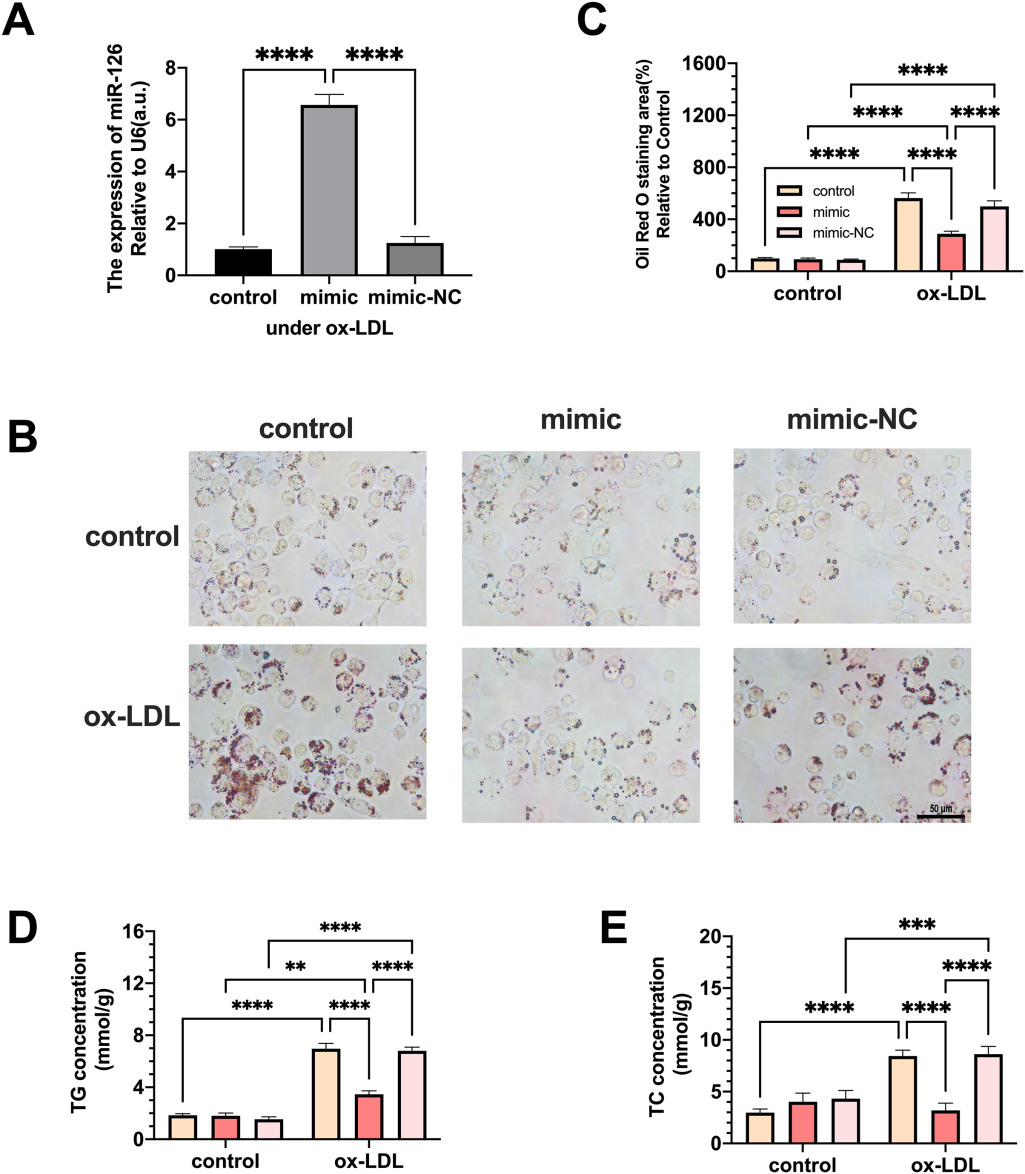
The effects of miR-126 transfection was evaluated in the expression of miR-126 and the change of foam cell area and lipid levels. (A) Levels of miR-126 after 50 μg/mL ox-LDL treatment and 100 nM mimic or mimic-NC transfection. Four asterisks (****) denotes *P* value < 0.0001 by one-way ANOVA analysis (*n* = 4, >1,000 cells per trial). (B) Oil red O staining area after 50 μg/mL ox-LDL treatment and 100 nM mimic or mimic-NC transfection was measured. Scale bar: 50 µm. (C) Quantification of lipid staining area from (B). Four asterisks (****) denote *P* value < 0.0001, by two-way ANOVA analysis (*n* = 6, >1,000 cells per trial). (D) TG and TC levels after 50 μg/mL ox-LDL treatment and 100 nM mimic or mimic-NC transfection. (E) Quantification of TG and TC levels from (D). Four asterisks (****) denote *P* value < 0.0001, three asterisks (***) denote *P* value < 0.001, and two asterisks (**) denote *P* value < 0.01 by two-way ANOVA analysis (*n* = 6, >1,000 cells per trial).

Macrophages were transfected with miR-126 mimic and negative control and the changes in lipid phagocytosis and apoptosis were investigated. As shown in Fig. 3A, miR-126 expression of macrophages was upregulated in the ox-LDL environment after transfection, implying that the mimic was successfully transfected. We found that the transfection greatly decreased foam cell development (Figs. 3B and 3C). In addition, TC and TG levels were strongly increased by ox-LDL and suppressed by the miR-126-5p mimic (Figs. 3D and 3E). These results suggest that miR-126 may regulate lipid metabolism to inhibit foam cell progression *in vitro*.

### MiR-126 influences M1 to M2 polarization and modulates inflammation

**Figure 4:**
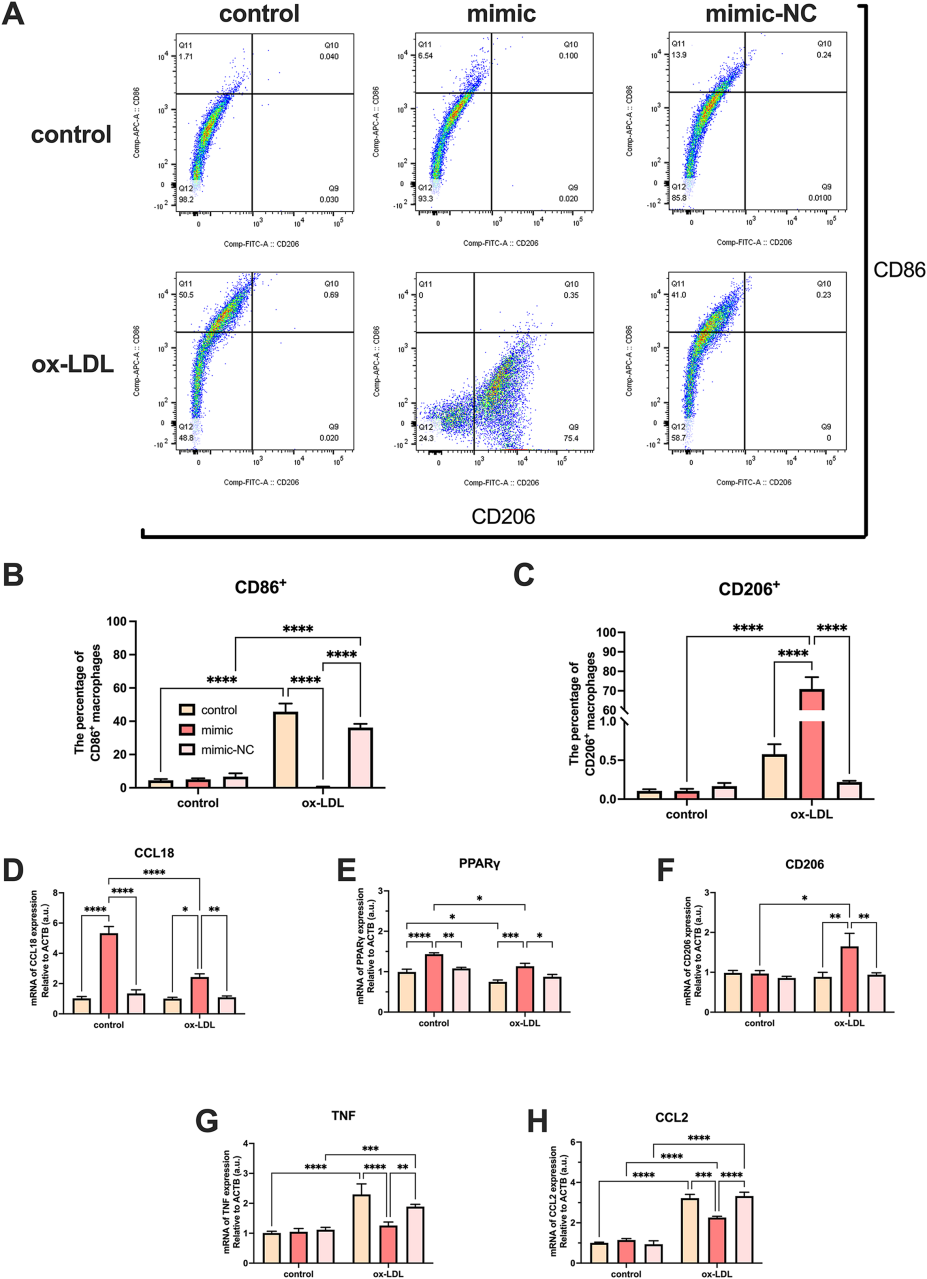
Ox-LDL was more likely to generate pro-inflammatory factors, favoring the M1 phenotype, while miR-126 reduced the synthesis of these factors, favoring the M2 phenotype. (A) Flow cytometry results after 50 μg/mL ox-LDL treatment and 100 nM mimic or mimic-NC transfection. (B and C) Quantification of CD86^+^ and CD206^+^ macrophages from (A). Four asterisks (****) denote *P* value < 0.0001 by two-way ANOVA analysis (*n* = 6, >1,000 cells per trial). (D−F) Quantification of CCL18, CD206, and PPAR γ (M2) mRNA levels. Four asterisks (****) denote *P* value < 0.0001, three asterisks (***) denote *P* value < 0.001, two asterisks (**) denote *P* value < 0.01, and one asterisk (*) denotes *P* value < 0.05 by two-way ANOVA analysis (*n* = 4, >1,000 cells per trial). (G−I) Quantification of TNF, and CCL2 (M1) mRNA levels. Four asterisks (****) denote *P* value < 0.0001, three asterisks (***) denote *P* value < 0.001, two asterisks (**) denote *P* value < 0.01, and one asterisk (*) denotes *P* value < 0.05 by two-way ANOVA analysis (*n* = 4, >1,000 cells per trial).

Ox-LDL-free groups appeared to have similar results and showed no statistical difference in the number of CD86^+^ and CD206^+^ cells. The population of CD86^+^ cells increased in the ox-LDL groups, but CD206^+^ did not. When ox-LDL and miR-126 both exist, CD206^+^ macrophages increased visually, and appeared to have little growth effect on CD86^+^ cells (Figs. 4A–4C). Thus, macrophages would not be affected by miR-126 if ox-LDL had not existed, as flow cytometry results have shown. The miR-126 raised the mRNA expression of M2 phenotype (Figs. 4D–4F) and downregulated that of M1 (Figs. 4G–4I). The mRNA levels indicated that the miR-126 could not stimulate inflammatory processes but was able to modulate ox-LDL-mediated inflammation. Ox-LDL boosted the expression of pro-inflammatory factors while miR-126 suppressed anti-inflammatory, which can facilitate the shift from M1 to M2 phenotype.

### MiR-126 promotes M1 to M2 polarization *via* the VEGFA/KLF4 signaling axis

**Figure 5:**
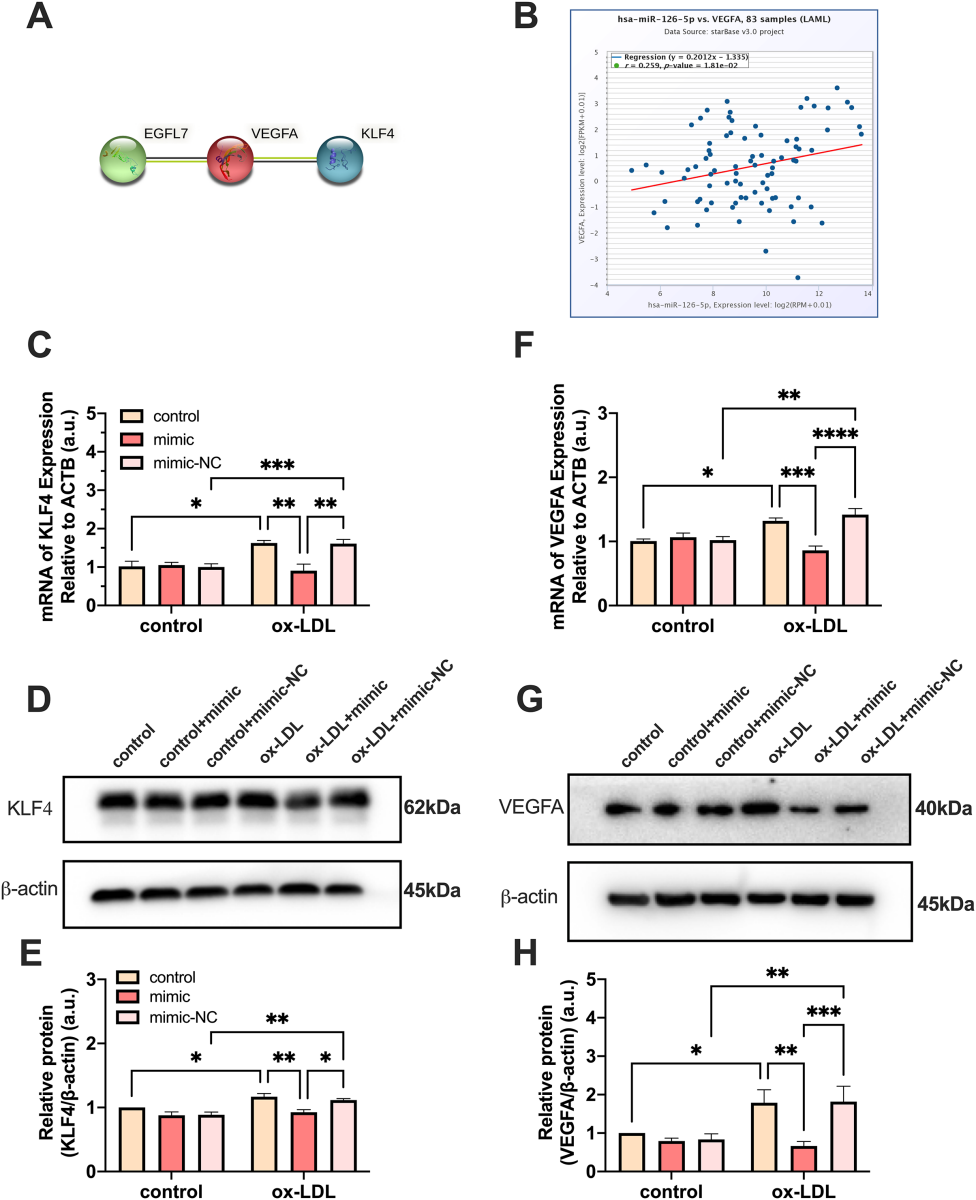
MiR-126 promoted macrophage phenotype to switch from M1 to M2 *via* VEGFA/KLF4 signaling. (A) PPI network generated with STRING website. (B) The miR-126-5p-VEGFA co-expression field from starBase. *P* = 0.0181. (C and F) Quantification of KLF4 and VEGFA mRNA levels. Four asterisks (****) denote *P* value < 0.0001, three asterisks (***) denote *P* value < 0.001, two asterisks (**) denote *P* value < 0.01, and one asterisk (*) denotes *P* value < 0.05 by one-way ANOVA analysis (*n* = 4, >1,000 cells per trial). (D and G) Western blots indicating total KLF4, VEGFA and β-Actin levels in whole cell lysates. (E and H) Quantification of KLF4 and VEGFA protein levels from (D and G). Three asterisks (***) denote *P* value < 0.001, two asterisks (**) denote *P* value < 0.01, and one asterisk (*) denotes *P* value < 0.05 by one-way ANOVA analysis (*n* = 4, >1,000 cells per trial).

PPI network analysis indicated a significantly high correlation between *EGFL7*, the host gene of miR-126, *VEGFA*, and *KLF4* (*P* = 0.0232) (Fig. 5A). The analysis showed that these proteins are biologically connected as a group with such an enrichment. The miR-126-5p-VEGFA co-expression field showed a significant positive correlation (*P* = 0.0181, *r* = 0.259) but barely had a direct connection with KLF4 (Fig. 5B). Expression of *VEGFA* and *KLF4* was enhanced by ox-LDL and inhibited by the miR-126-5p mimic at the transcriptional level (Figs. 5C and 5F). Furthermore, the reduction in the mRNA levels of *VEGFA* and *KLF4* was consistent with the downregulation in the protein levels (Figs. 5D and 5E, 5G and 5H). These findings revealed that miR-126 may promote the macrophage phenotype to switch from M1 to M2, based on the *in vitro* expression of VEGFA/KLF4.

#### Both miR-126 and ox-LDL promoted endothelial cell migration and miR-126 inhibited the migration of endothelial cells induced by ox-LDL

**Figure 6:**
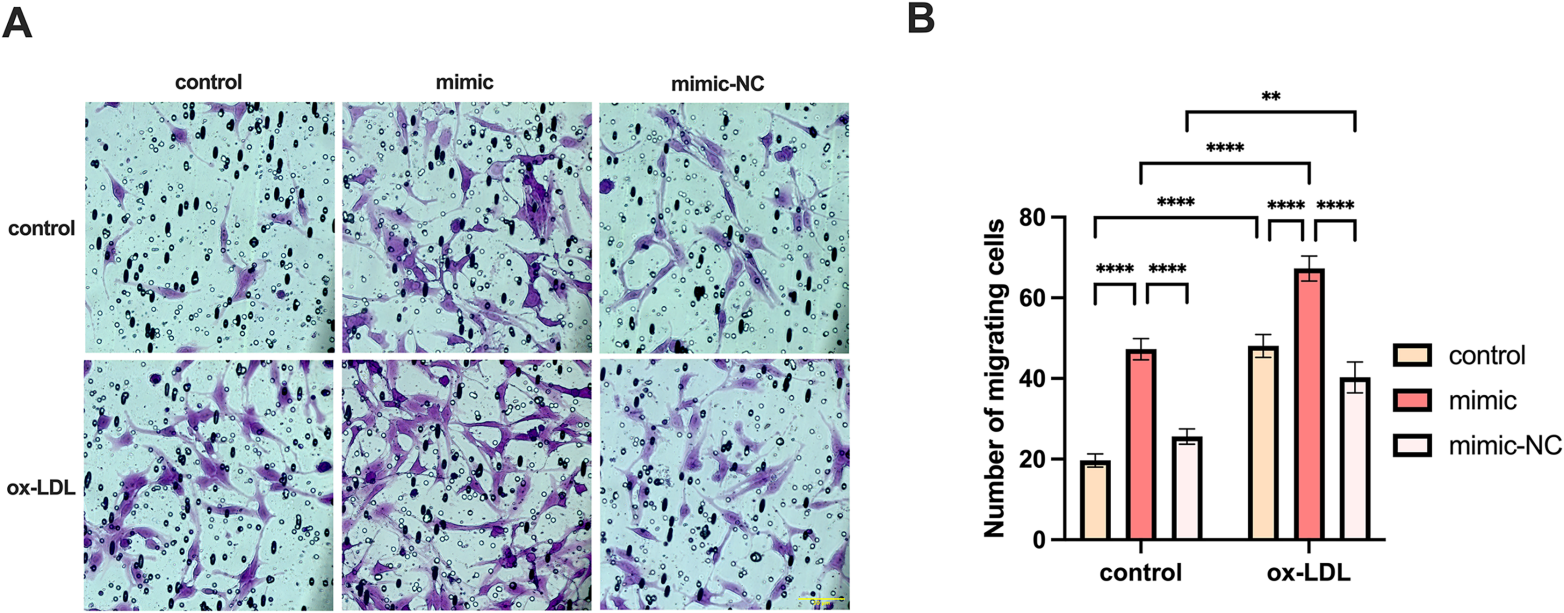
Both miR-126 and ox-LDL promoted endothelial cell migration and miR-126 inhibited the migration of endothelial cells induced by ox-LDL. (A) The crystal violet staining of the chambers after 50 μg/mL ox-LDL treatment and 100 nM mimic or mimic-NC transfection. Scale bar: 50 µm. (B) Quantification of cell number across the membranes from (B). Four asterisks (****) denote *P* value < 0.0001, two asterisks (**) denote *P* value < 0.01 by two-way ANOVA analysis (*n* = 5, >1,000 cells per trial).

We co-cultured macrophages with human umbilical vein endothelial cells to measure the migration of endothelial cells. The migration of endothelial cells in the ox-LDL group was 2.44-fold higher than those in the control group, while the migration in the miR-126 group was 2.4-fold higher than those in the control group. However, the migration of endothelial cells in the ox-LDL + miR-126 group was 1.42 times that of the miR-126 group (Figs. 6A and 6B). This data indicated that miR-126 and ox-LDL were both capable of promoting endothelial cell migration and miR-126 inhibited the migration of endothelial cell induced by ox-LDL.

#### MG-132 increased macrophages lipid uptake by activating AP-1/VEGFA/KLF4 axis

**Figure 7:**
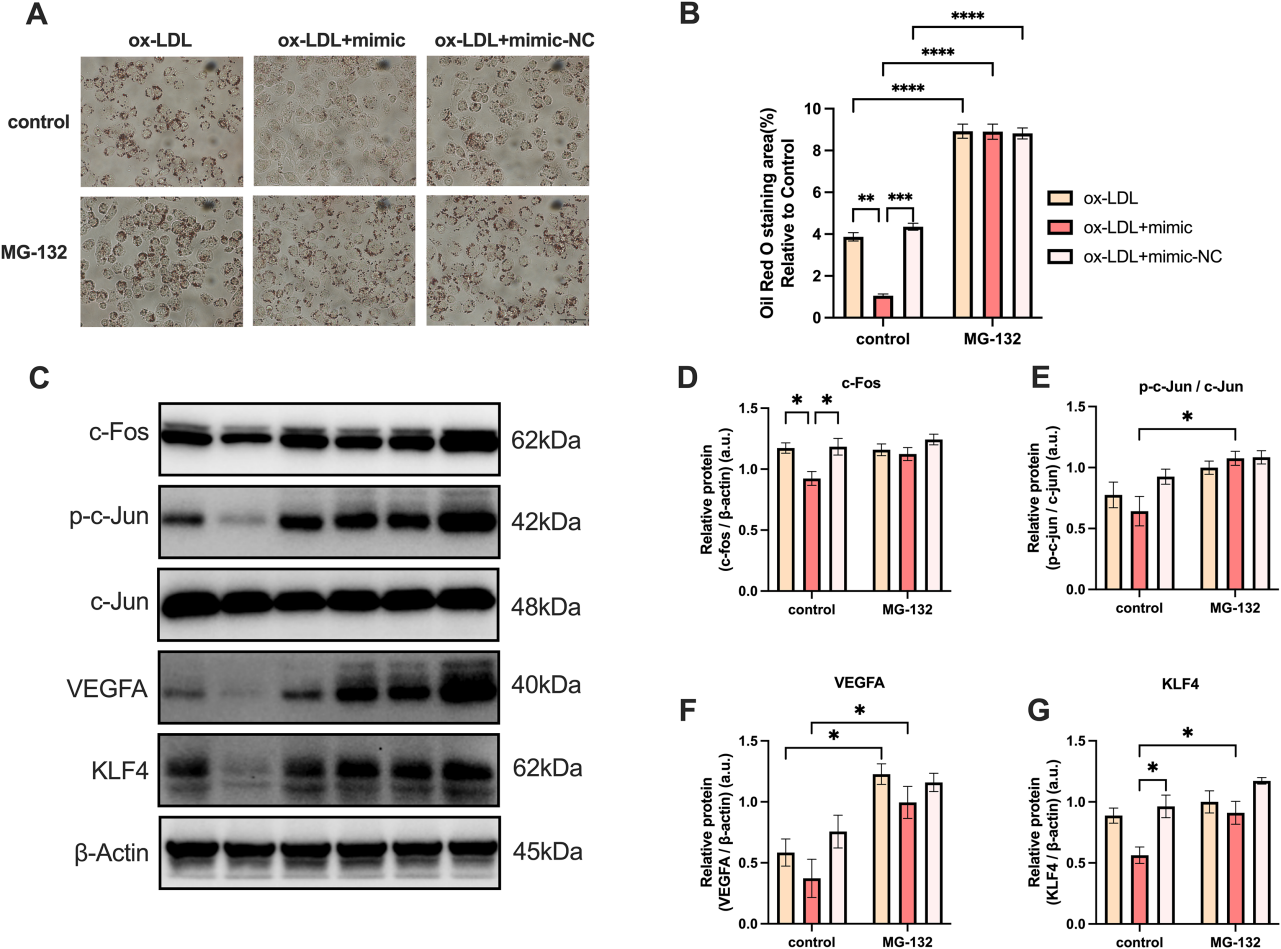
MG-132 increased macrophages lipid uptake by activating AP-1/VEGFA/KLF4 axis. (A) Oil red O staining area after 50 μg/mL ox-LDL treatment and 100 nM mimic or mimic-NC transfection for 4 h while MG-132 treatment was measured. Scale bar: 50 µm. (B) Quantification of lipid staining area from (A). Four asterisks (****) denote *P* value < 0.0001, three asterisks (***) denote *P* value < 0.001, two asterisks (**) denote *P* value < 0.01, by two-way ANOVA analysis (*n* = 6, >1,000 cells per trial). (C) Western blots of total c-Fos, p-c-Jun, c-Jun, VEGFA, KLF4, and β-Actin levels in whole cell lysates. (D−G) Quantification of c-Fos, p-c-Jun, c-Jun, VEGFA, KLF4, and β-Actin protein levels from (C). An asterisk (*) denotes *P* value < 0.05 by two-way ANOVA analysis (*n* = 4, >1,000 cells per trial).

MG-132 has been previously demonstrated to be an activator of the Protein-1 (AP-1) family members c-Fos and c-Jun (Li, Zhang & Olumi, 2007), which activates the AP-1/VEGFA/KLF4 axis. As illustrated in Fig. 7, macrophage lipid uptake reduced in the ox-LDL + miR-126 group in comparison to the ox-LDL group alone. The addition of MG-132, however, was able to restore lipid uptake levels that were decreased by ox-LDL with miR-126 (Figs. 7A and 7B). The data also revealed that miR-126 significantly decreased the level of c-Fos, when compared with that in the ox-LDL group (Figs. 7C and 7D). MG-132 increased the phosphorylation level of c-Jun in the presence of both ox-LDL and miR-126 (Figs. 7C and 7E). The utilization of MG-132 increased the levels of VEGFA (Figs. 7C and 7F). miR-126 was found to significantly decrease the levels of KLF4. However, the application of MG-132 significantly increased the levels of KLF4 (Figs. 7C and 7G). Based on these findings, we propose that MG-132 increases macrophages lipid uptake by activating the AP-1/VEGFA/KLF4 axis.

## Discussion

Foam cells, which are critical to the development and extension of necrotic cores in atherosclerotic plaques, are formed when ox-LDL is recognized and taken up by scavenger receptor macrophages (Ball et al., 1995). In this study, we observed that upon 25 and 50 μg/mL ox-LDL exposure, macrophages exhibited enhanced phagocytic and apoptotic activity, which had no statistical difference. However, the late apoptosis (Q2) rate of the 50 µg/mL group sharply increased, whereas the early apoptosis (Q1) rate seemed to be relatively lower when compared to the 25 µg/mL group (Fig. 1C). We chose 50 µg/mL ox-LDL dose because a higher dose helped macrophages transform into foam cells with greater ease, and the apoptosis ratio was almost about 10% at this dose which ensured the effective implementation of the experiment. We justified this with a possible saturation of the phagocytic capacity of foam cells at 50 μg/mL for 48 h. Foam cells may deplete ox-LDL with the increase in exposure time, especially over 48 h. Taken together, 50 μg/mL ox-LDL and 24 h are comparatively the most appropriate parameters for inducing apoptosis of differentiated macrophages. Our findings are consistent with those of other studies (Wang et al., 2019; Zhong et al., 2020).

Endothelial miR-126 levels decline may be associated with plaque formation (Mondadori dos Santos et al., 2015). Deficiency of miR-126 causes a deterioration of the endothelial tube hierarchy, hemorrhages, and decreased EC proliferation and migration in the blood arteries of mice (Wang et al., 2008). The serum miR-126 level was dramatically lowered in CAD individuals with higher LDL and small dense low-density lipoprotein (sdLDL) cholesterol levels (Mishra et al., 2021; Sun et al., 2012). However, circulating miR-126 levels were significantly elevated when LDL and sdLDL were higher in individuals with risk factors of CAD, although this was not observed in those with clinically confirmed CAD (Mishra et al., 2021; Sun et al., 2012). We suspected that ox-LDL played a role in the elevation of miR-126. Although the function of endothelial miR-126 is extensively recognized, its relevance in macrophages (particularly in AS) has not been extensively investigated.

Our results demonstrate that the miR-126-5p mimic can be successfully transfected into macrophages and suppresses foam cell development by lowering the levels of TC and TG. Pro-inflammatory factors like *TNFA*, and *CCL2* were elevated, while anti-inflammatory factors like *CCL18*, *PPAR γ*, and *CD206* decreased by miR-126-5p. Additionally, CD206^+^ macrophages increased visually by miR-126-5p, and CD86^+^ cells appeared to have little growth effects. The miR-126 has also been found to regulate macrophage recruitment and pro-inflammatory cytokines levels reduction (Wu et al., 2022), which proves the technicality of our study.

The starBase database in Pan-Cancer shows that miR-126-5p correlates with VEGFA in the miRNA-target co-expression field. *EGFL7*, as the host gene of miR-126, has interactions with VEGFA in the PPI network. Moreover, VEGFA and KLF4 are highly correlated with each other. Our results indicated that VEGFA and KLF4 expressions are enhanced by ox-LDL and inhibited by the miR-126-5p mimic at the protein and transcript levels. Therefore, we speculate that the formation of VEGFA and KLF4 is closely related to the role of miR-126 *in vitro*.

Several studies have reported that miR-126 promotes EC migration (Chistiakov, Orekhov & Bobryshev, 2016; Wang et al., 2008; Zhou et al., 2016), and it is widely accepted that ox-LDL promotes inflammation and EC migration (Libby, 2021; Song et al., 2020; Wolf & Ley, 2019). Our findings are consistent with these reports. In our study, miR-126 exhibited anti-inflammatory effects on foam cells and promoted the expression of anti-inflammatory genes such as *PPAR γ*. Therefore, we initially hypothesized that miR-126 would inhibit the migration of ECs co-cultured with foam cells. However, as shown in Fig. 6, this was not the case. According to Fig. 3, this apparent discrepancy can be explained by the fact that miR-126 promotes the expression of *PPAR γ* in macrophages. *PPAR γ* has been shown in other studies (Halabi & Sigmund, 2005; Uddin et al., 2019; Werner et al., 2014) to promote EC proliferation and migration. Thus, the increased EC migration in Fig. 6 is the result of the combined effects of ox-LDL and miR-126, with the former promoting inflammation and the latter activating macrophage *PPAR γ* expression. Although both ox-LDL and miR-126 promote EC migration, the pro-inflammatory effects of ox-LDL and the anti-inflammatory effects of miR-126 are opposite. We observed that the EC migration promoted by ox-LDL+miR-126 was not greater than the sum of the EC migration promoted by miR-126 and ox-LDL alone, and instead displayed ‘1+1 < 2’. Therefore, there may be a counterbalancing effect of inflammation and anti-inflammation in the ox-LDL+miR-126 group. Based on these findings, we propose that miR-126 and ox-LDL were both capable of promoting endothelial cell migration and miR-126 inhibited the migration of endothelial cell induced by ox-LDL. There are certain limitations in our study: due to budget constraints, we were forced to give up the detection of *PPAR γ* content in macrophage culture supernatant, and the relationship between miR-126 and *PPAR γ* remains unknown. However, there are many literature reports that macrophages express and detect *PPAR γ* in their supernatant (Bouhlel et al., 2007; Hernandez-Quiles, Broekema & Kalkhoven, 2021; Lee et al., 2022).

We used the activator of VEGFA upstream gene AP-1, MG-132, to indirectly activate the expression of VEGFA, and the expression of KLF4 also significantly increased, further explaining the relationship between VEGFA and KLF4 which was not described in Fig. 5. At the same time, to prove the accuracy of our experiment, we also detected c-Fos and c-Jun in the AP-1 family, and detected the phosphorylation level of c-Jun, thus it can be concluded that MG-132 can indeed activate the expression of the AP-1 family. Unfortunately, we found that the inhibition effect of miR-126 was limited and could not offset the excitatory effect of MG-132.

AS is the build-up of plaques in the artery walls due to chronic inflammation, and it is primarily caused by LDL. Our findings revealed that 50 µg/mL dose ox-LDL for 24 h leads to the activation of the phagocytic and apoptotic function of macrophages, and induces the formation of foam cells. However, mimic treatment resulted in a decrease in foam cell formation and production of pro-inflammatory mRNA levels, most likely due to the transition from the M1 to M2 phenotype. PPI network analysis and miR-126-5p-VEGFA co-expression field images from starBase revealed that miR-126 may promote M1 switching to M2 *in vitro via* the VEGFA/KLF4 signaling axis. Furthermore, the reduction in VEGFA levels and KLF4 proteins was consistent with the decrease in transcript levels. MiR-126 also promotes EC migration by activating macrophage *PPAR γ* expression and effectively suppressing macrophage inflammation. MG-132 indirectly activated the expression of VEGFA, and the expression of KLF4 also significantly increased, which indicates a direct or indirect relationship between VEGFA and KLF4. However, what we did is only on one type of macrophage, further research is required in this direction to explore human primary macrophages and associate them with CAD patients.

## Conclusions

In conclusion, miR-126 inhibited foam cell formation and apoptosis. Moreover, our data highlighted that miR-126 promotes macrophage polarization toward the M2 phenotype by downregulating VEGFA and KLF4. The above results provide a new perspective for revealing macrophage polarization from the viewpoint of miR-126 transfection in the ox-LDL environment.

## Supplemental Information

10.7717/peerj.15180/supp-1 Supplemental Information 1 Full-length uncropped blots of Figure 5 and all replicates.

10.7717/peerj.15180/supp-2 Supplemental Information 2 Full-length uncropped blots of Figure 7 and all replicates.

10.7717/peerj.15180/supp-3 Supplemental Information 3 The procedure of Transwell.

## Additional Information and Declarations

### Data Availability

The following information was supplied regarding data availability: The data is available at Zenodo: Qiang Liu. (2022). miR-126 promotes M1 to M2 macrophage phenotype switching *via* VEGFA and KLF4 (formal version-2) [Data set]. Zenodo. https://doi.org/10.5281/zenodo.6774693.
