# Supplementary material for: miR-126 promotes M1 to M2 macrophage phenotype switching via VEGFA and KLF4
Source: PeerJ. 2023 Mar 31;11:e15180. doi: 10.7717/peerj.15180 (PMC10069419; doi:10.7717/peerj.15180)
Supplement: Supplemental Information 1 [file peerj-11-15180-s001.zip › Figure5 full-length uncropped blots /WB.pdf]

KLF4 | **β-actin**

contrast-1

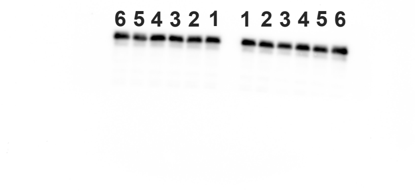

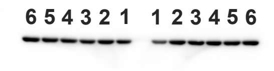

contrast-8

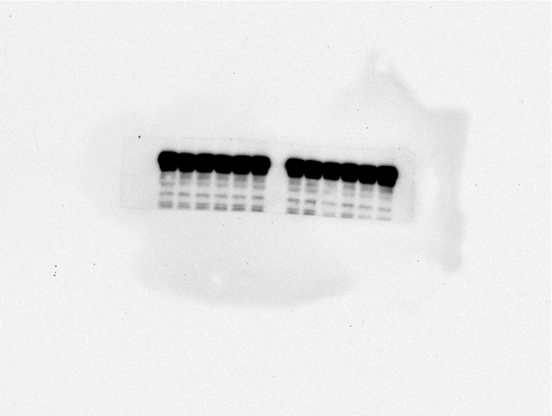

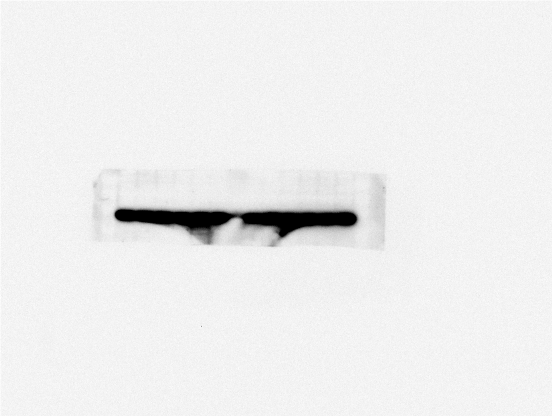

VEGFA | **β-actin**

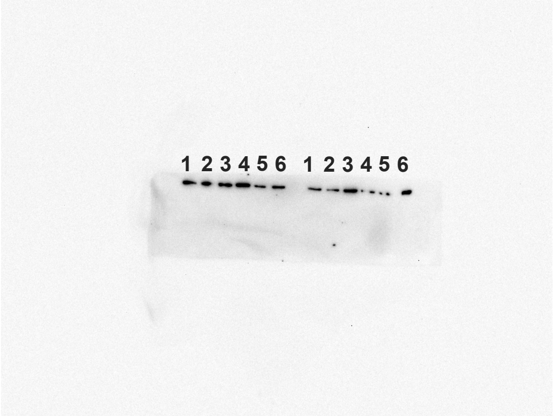

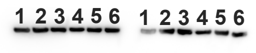

group:
1=control,
2=control+mimic,
3=control+mimic–NC,
4=ox–LDL, 5=ox–LDL+mimic,
6=ox–LDL+mimic–NC

Original file of this β-**actin** was provided with this PDF file. Other documents have shown the situation of uncutting PVDF film.
