# Supplementary figures and images for: miR-126 promotes M1 to M2 macrophage phenotype switching via VEGFA and KLF4
Source: PeerJ. 2023 Mar 31;11:e15180. doi: 10.7717/peerj.15180 (PMC10069419; doi:10.7717/peerj.15180)

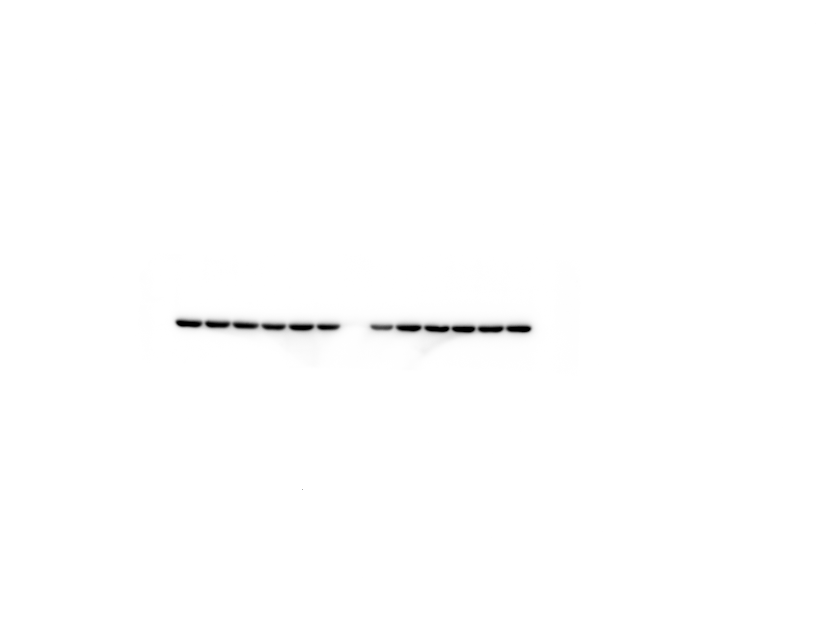

Supplement: Supplemental Information 1 [file peerj-11-15180-s001.zip › Figure5 full-length uncropped blots /2021-10-16b-actin-contrast_1.png]

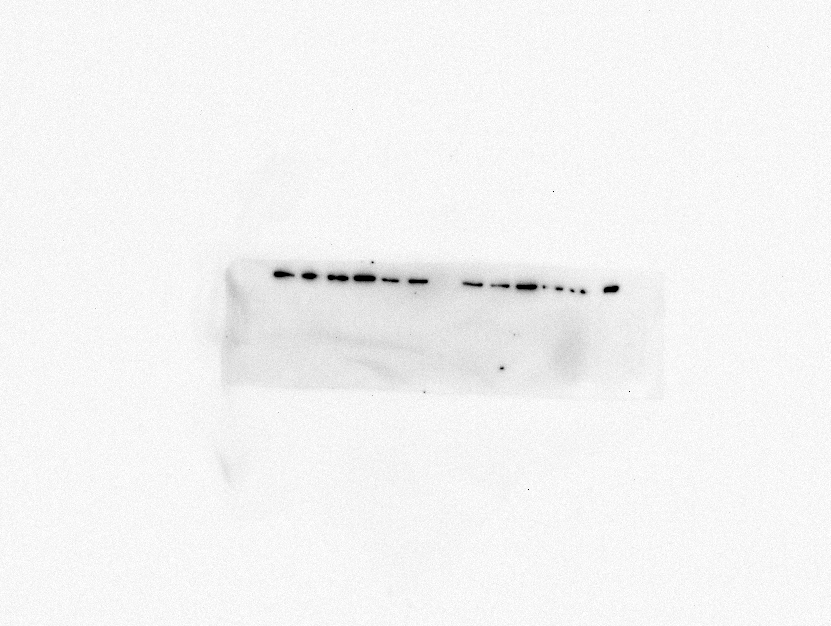

Supplement: Supplemental Information 1 [file peerj-11-15180-s001.zip › Figure5 full-length uncropped blots /2021-10-30sxy-vegfa.png]

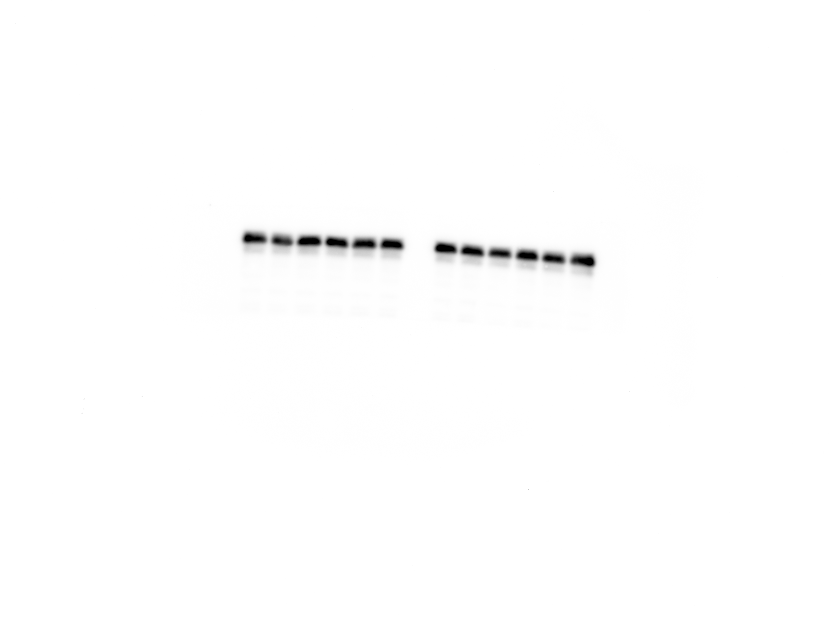

Supplement: Supplemental Information 1 [file peerj-11-15180-s001.zip › Figure5 full-length uncropped blots /2021-10-15KLF4-contrast_1.png]

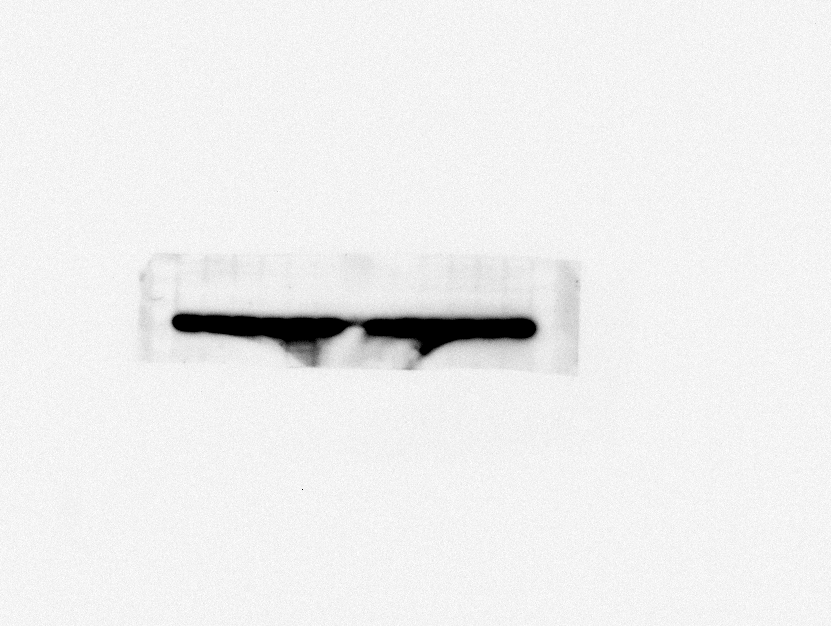

Supplement: Supplemental Information 1 [file peerj-11-15180-s001.zip › Figure5 full-length uncropped blots /2021-10-16b-actin-contrast_8.png]

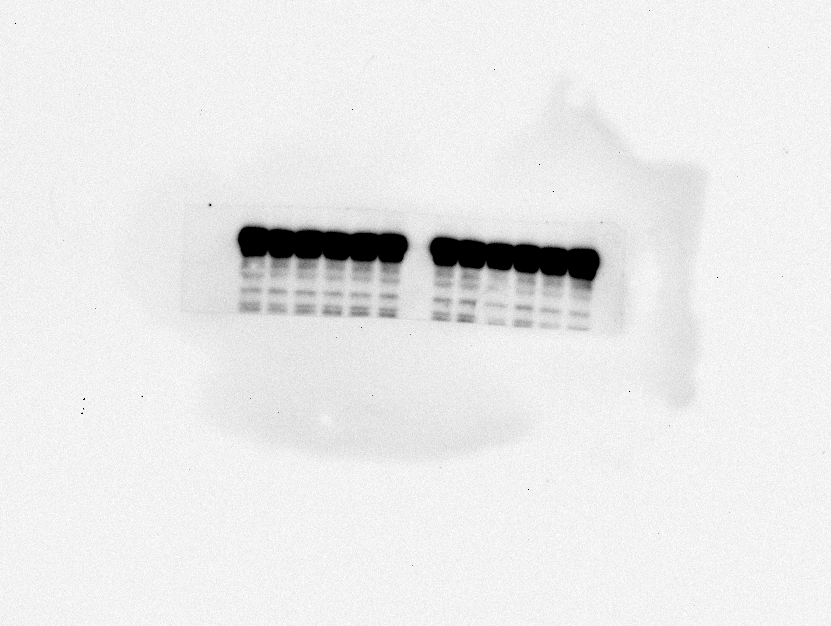

Supplement: Supplemental Information 1 [file peerj-11-15180-s001.zip › Figure5 full-length uncropped blots /2021-10-15KLF4-contrast_8.png]

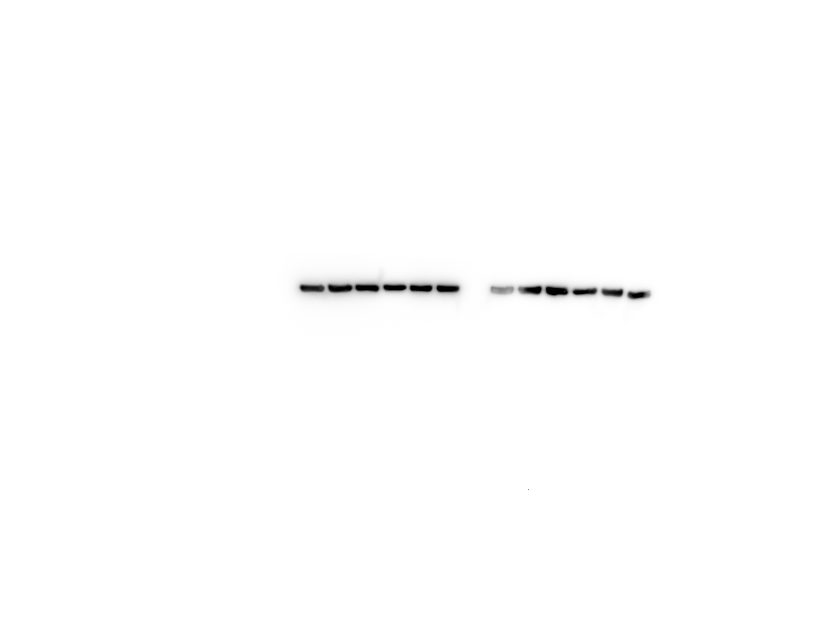

Supplement: Supplemental Information 1 [file peerj-11-15180-s001.zip › Figure5 full-length uncropped blots /2021-10-31Sxy-bactin.png]

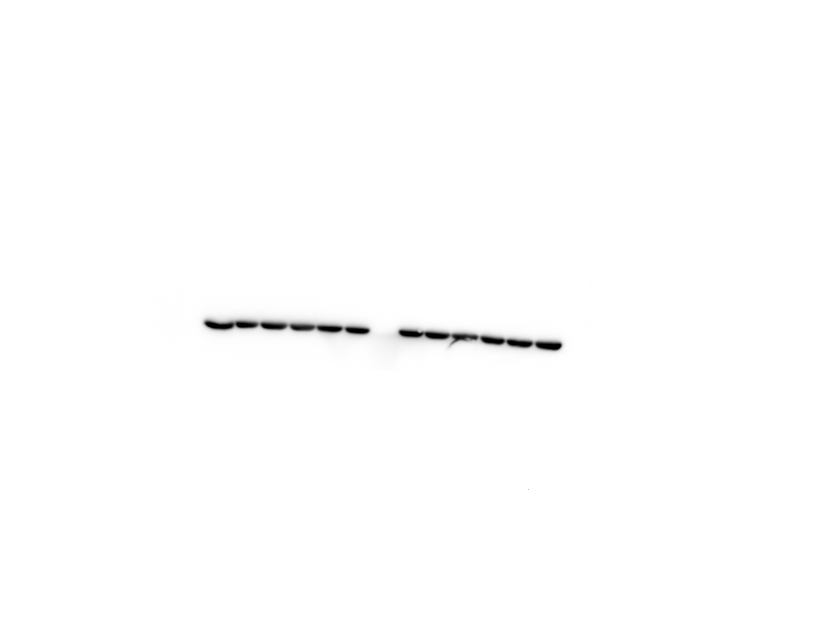

Supplement: Supplemental Information 1 [file peerj-11-15180-s001.zip › Replicates/KLF4+b-actin/2021-10-17b-actin-2.png]

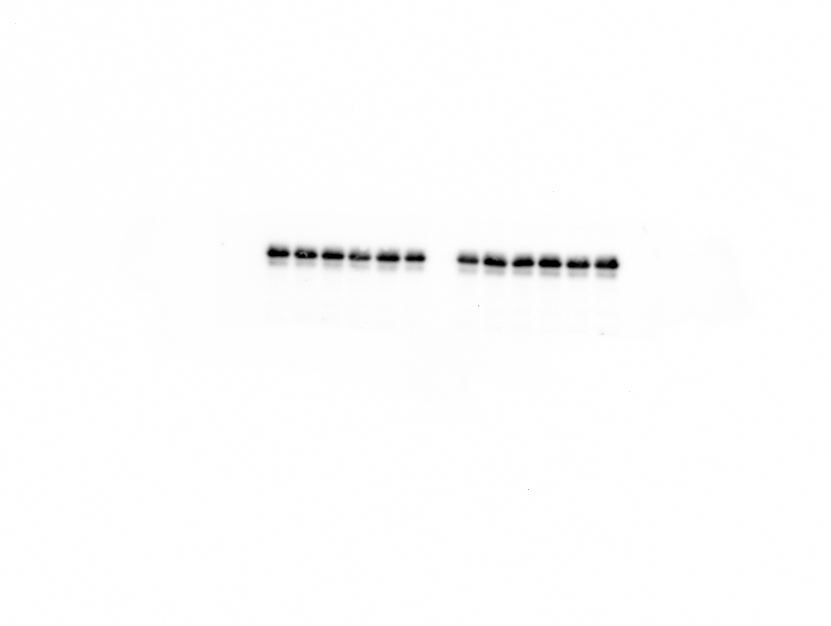

Supplement: Supplemental Information 1 [file peerj-11-15180-s001.zip › Replicates/KLF4+b-actin/2021-10-17KLF4-3.png]

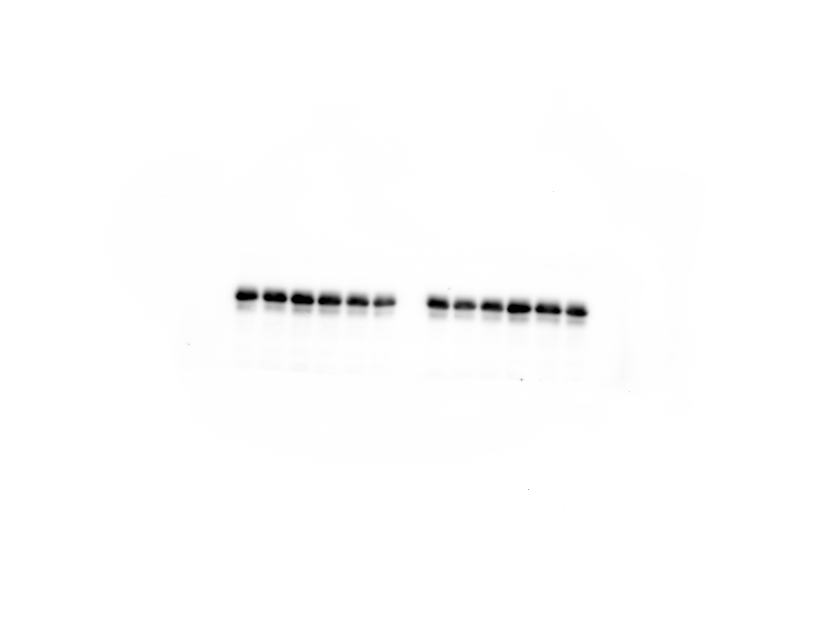

Supplement: Supplemental Information 1 [file peerj-11-15180-s001.zip › Replicates/KLF4+b-actin/2021-10-16KLF4-2.png]

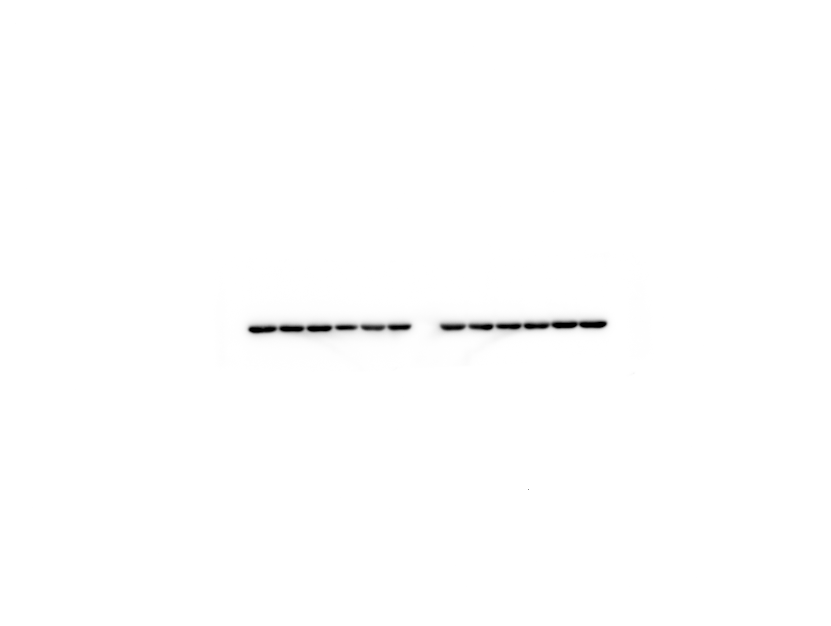

Supplement: Supplemental Information 1 [file peerj-11-15180-s001.zip › Replicates/KLF4+b-actin/2021-10-18bactin-3.png]

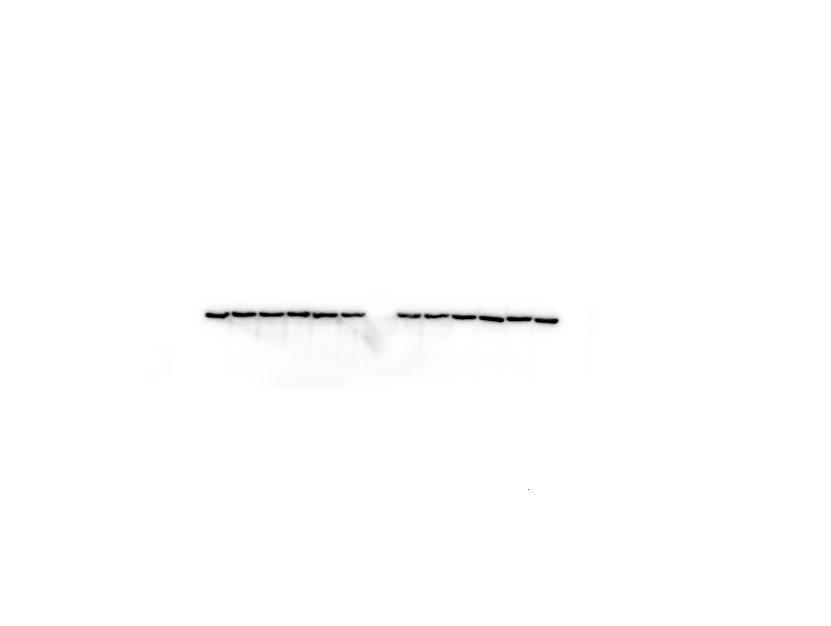

Supplement: Supplemental Information 1 [file peerj-11-15180-s001.zip › Replicates/VEGFA+b-actin/2021-11-02b-actin-3.png]

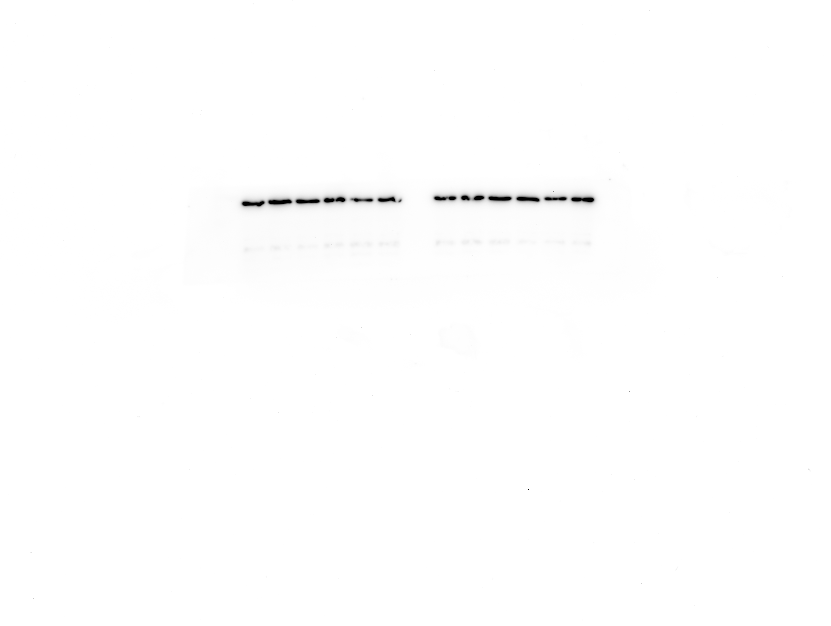

Supplement: Supplemental Information 1 [file peerj-11-15180-s001.zip › Replicates/VEGFA+b-actin/2021-11-01VEGFA-3.png]

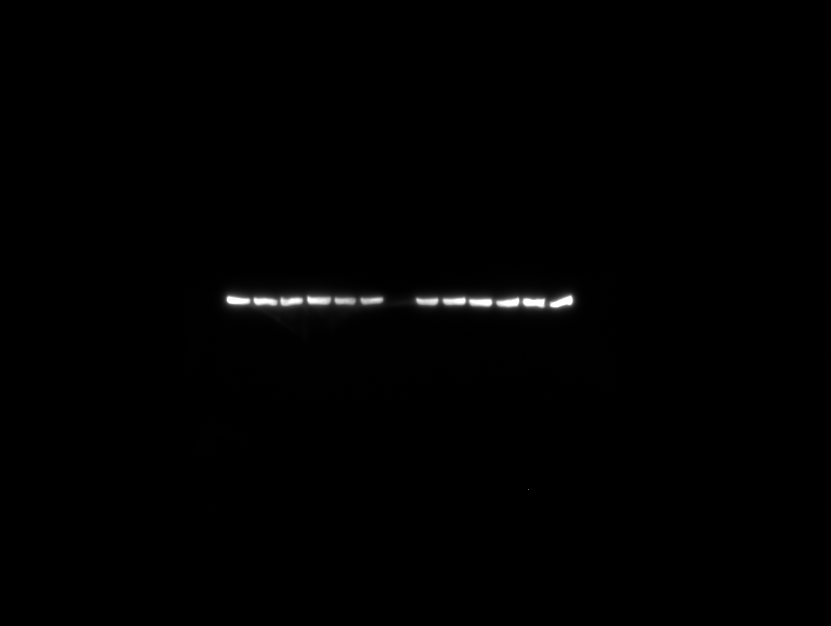

Supplement: Supplemental Information 1 [file peerj-11-15180-s001.zip › Replicates/VEGFA+b-actin/2021-11-03b-actin-4.png]

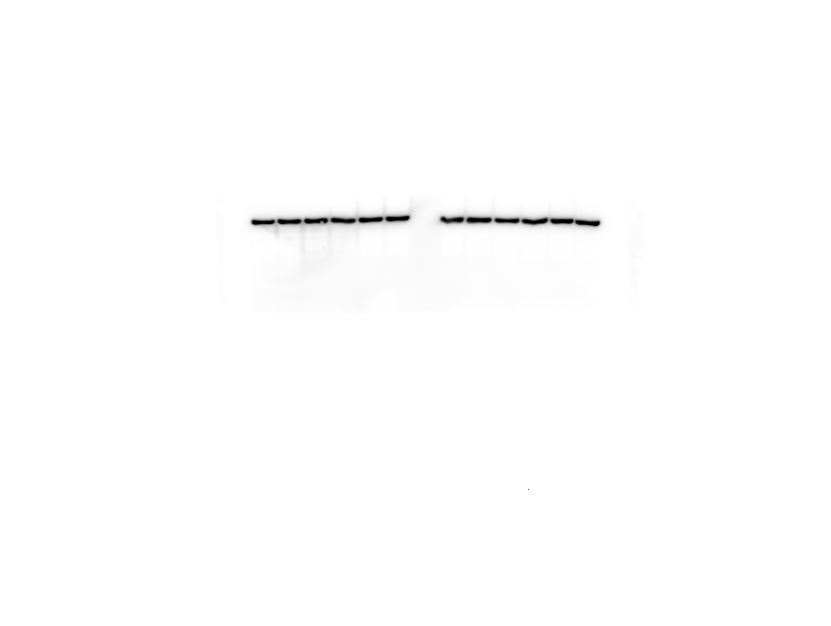

Supplement: Supplemental Information 1 [file peerj-11-15180-s001.zip › Replicates/VEGFA+b-actin/2021-11-01b-actin-2.png]

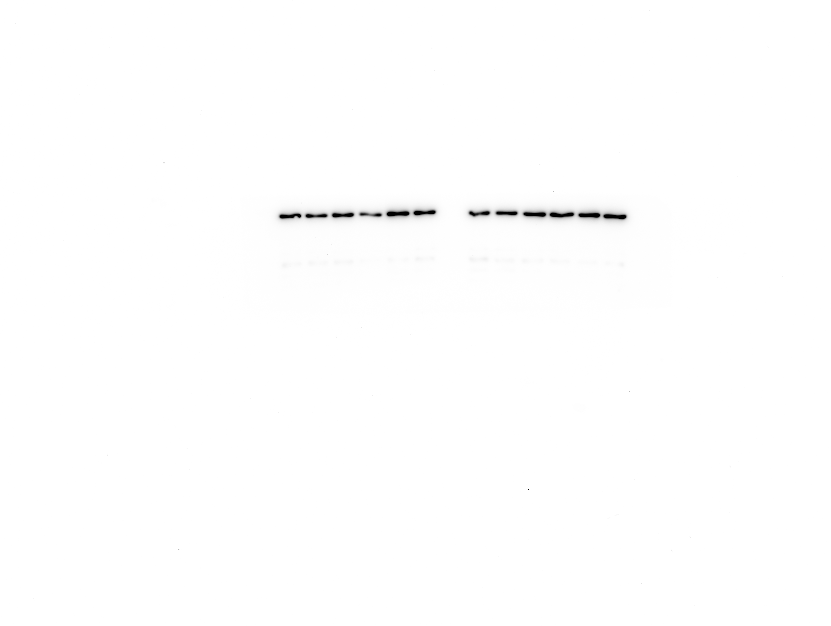

Supplement: Supplemental Information 1 [file peerj-11-15180-s001.zip › Replicates/VEGFA+b-actin/2021-11-02VEGFA-4.png]

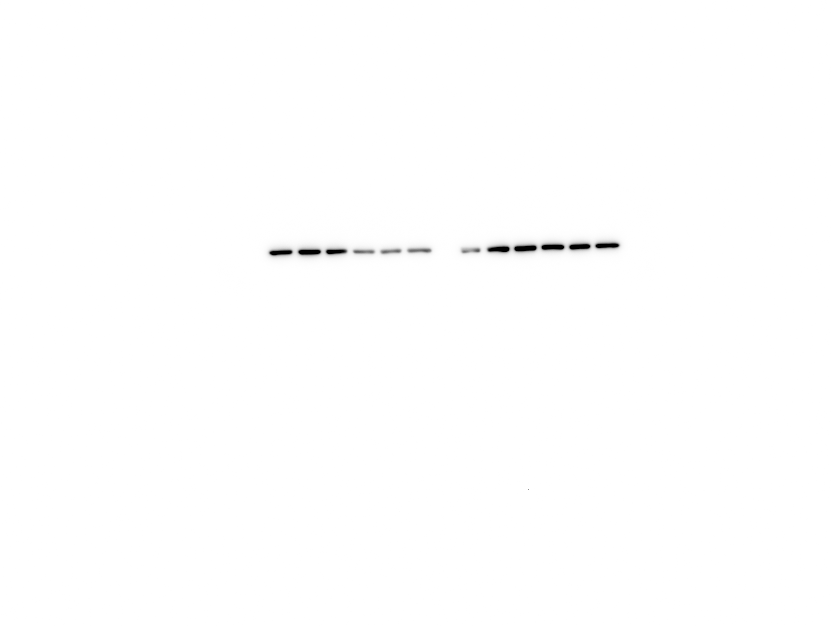

Supplement: Supplemental Information 1 [file peerj-11-15180-s001.zip › Replicates/VEGFA+b-actin/2021-10-31VEGFA-2.png]

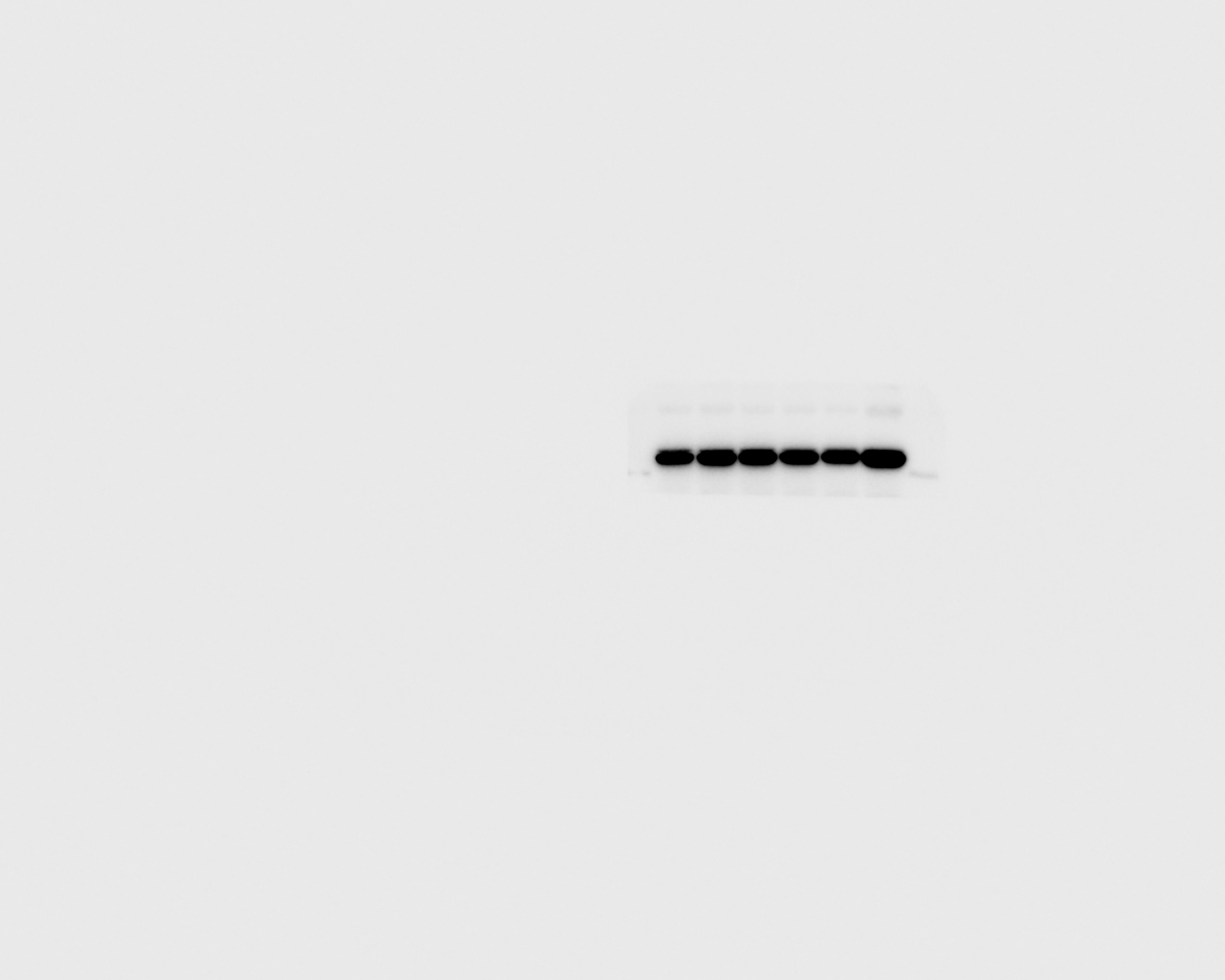

Supplement: Supplemental Information 2 [file peerj-11-15180-s002.zip › Figure7 full-length uncropped blots and replicates/Actin-A.tif]

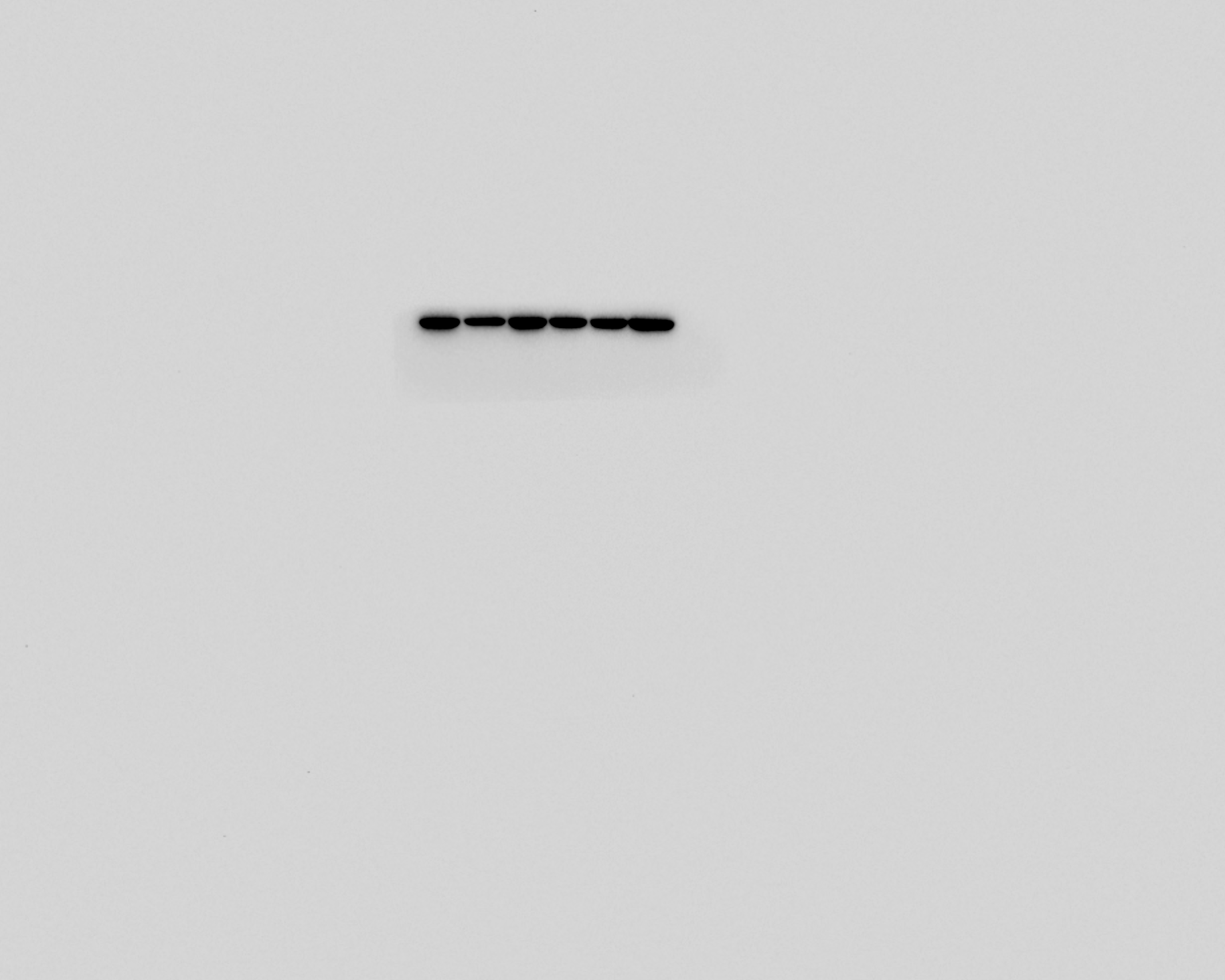

Supplement: Supplemental Information 2 [file peerj-11-15180-s002.zip › Figure7 full-length uncropped blots and replicates/p-c-jun-A.tif]

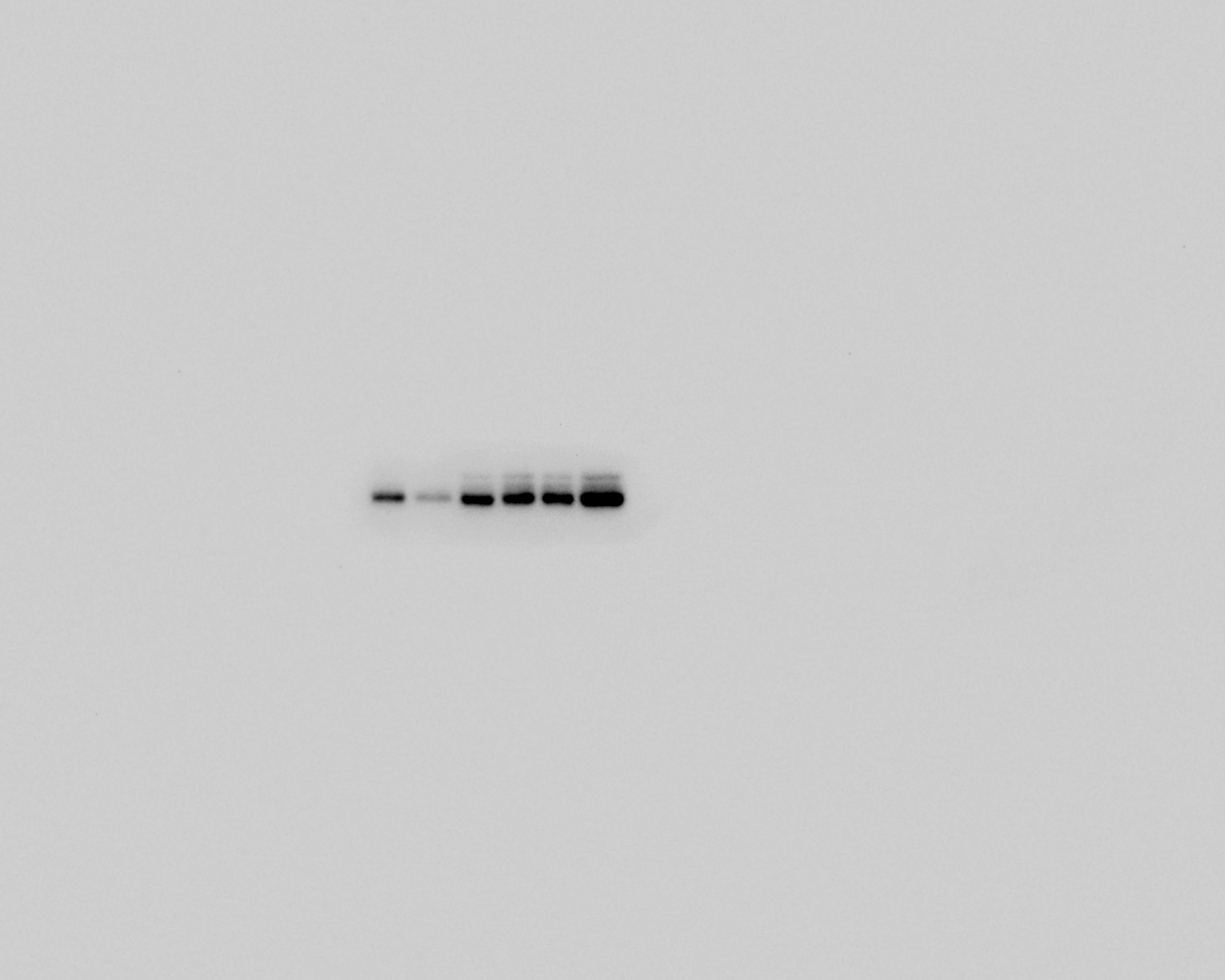

Supplement: Supplemental Information 2 [file peerj-11-15180-s002.zip › Figure7 full-length uncropped blots and replicates/p-c-jun-C.tif]

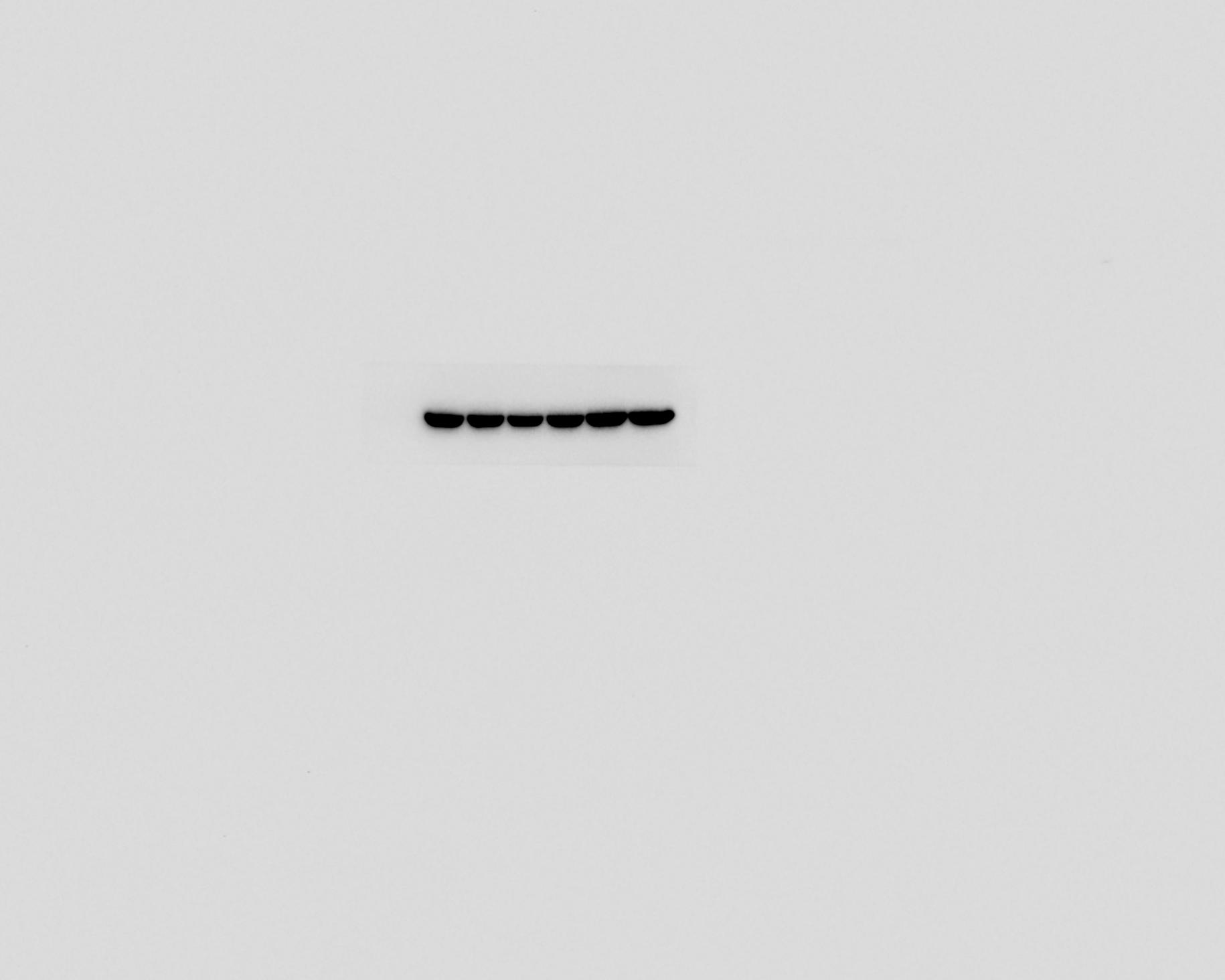

Supplement: Supplemental Information 2 [file peerj-11-15180-s002.zip › Figure7 full-length uncropped blots and replicates/Actin-B.tif]

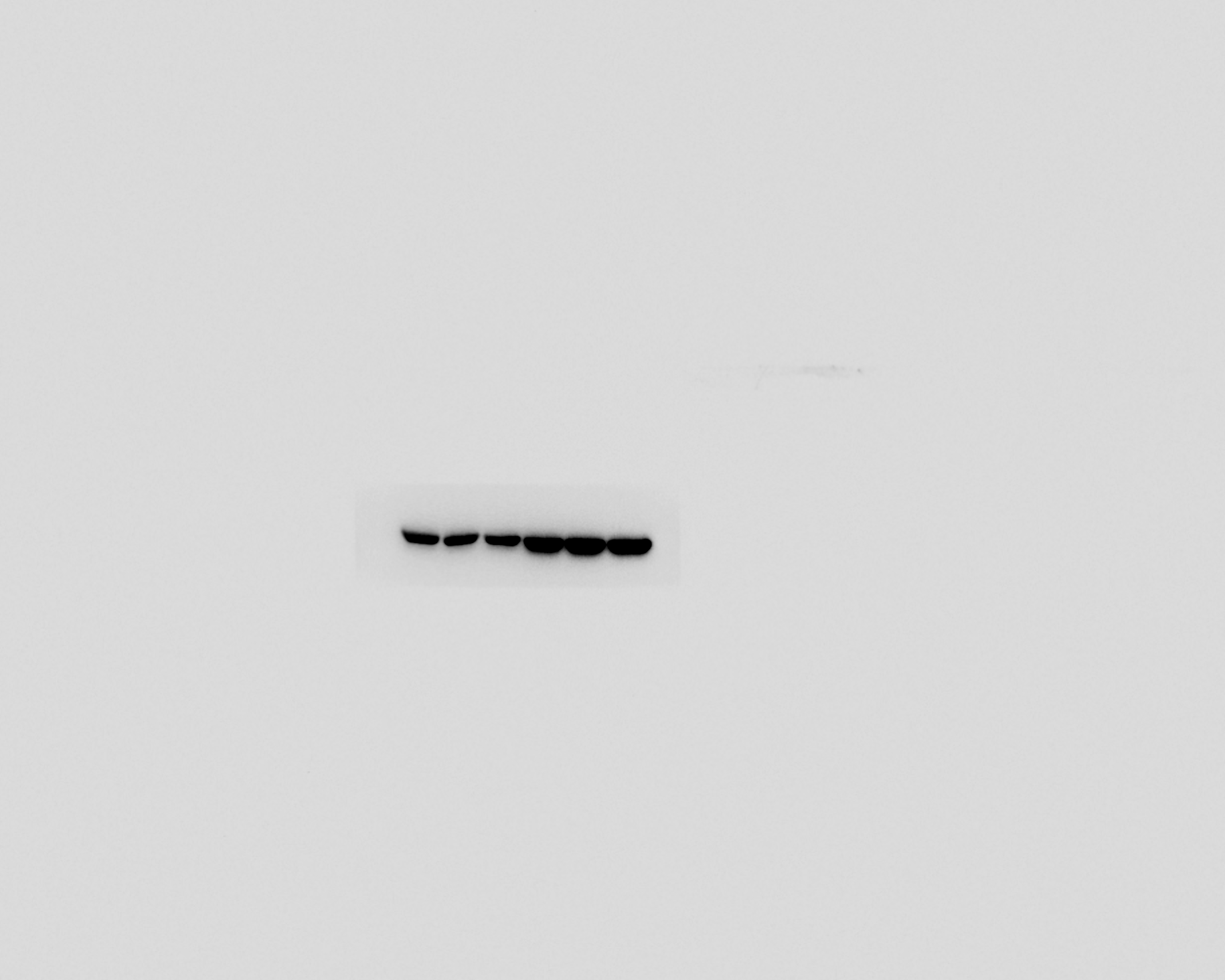

Supplement: Supplemental Information 2 [file peerj-11-15180-s002.zip › Figure7 full-length uncropped blots and replicates/p-c-jun-B.tif]

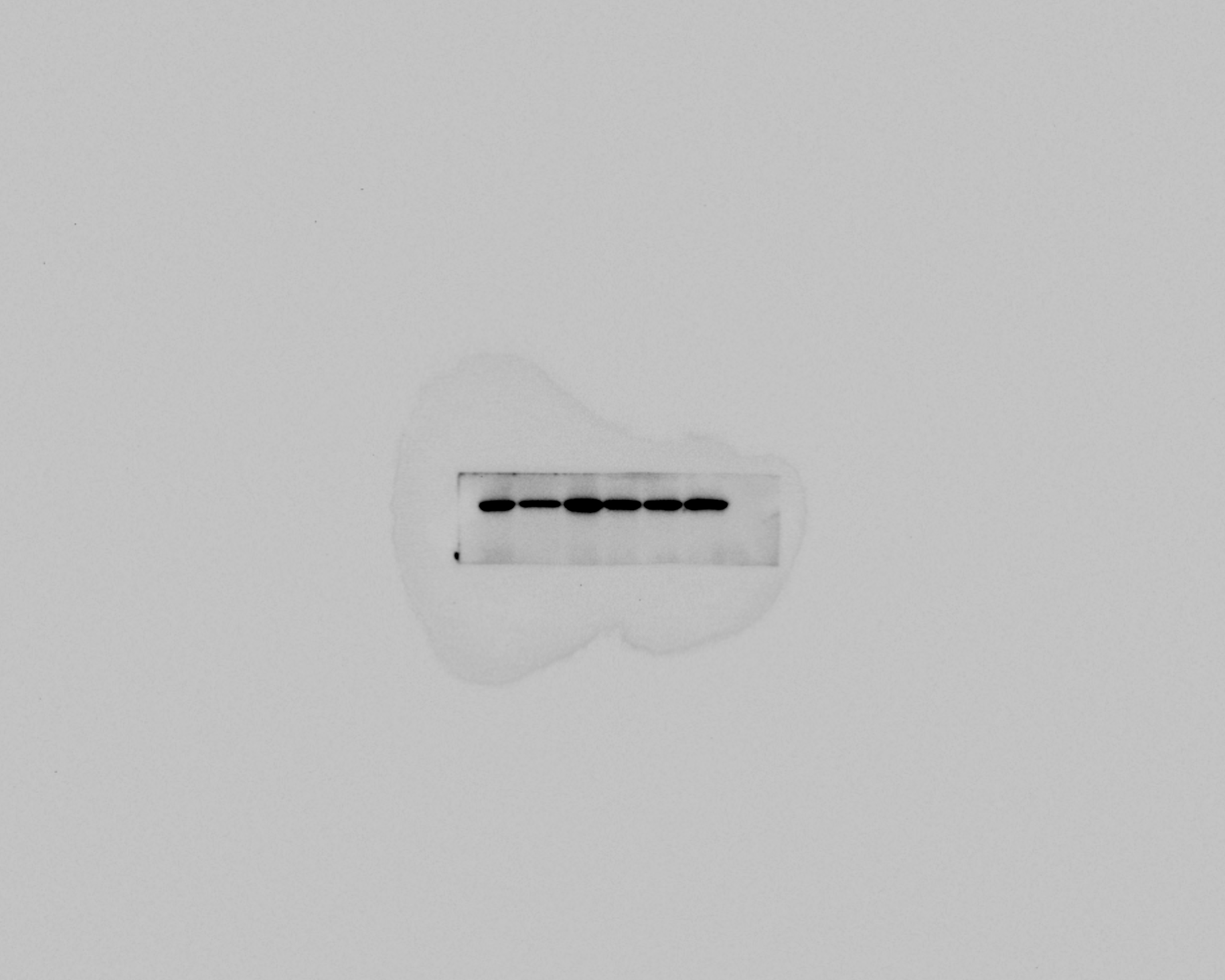

Supplement: Supplemental Information 2 [file peerj-11-15180-s002.zip › Figure7 full-length uncropped blots and replicates/p-c-jun-D.tif]

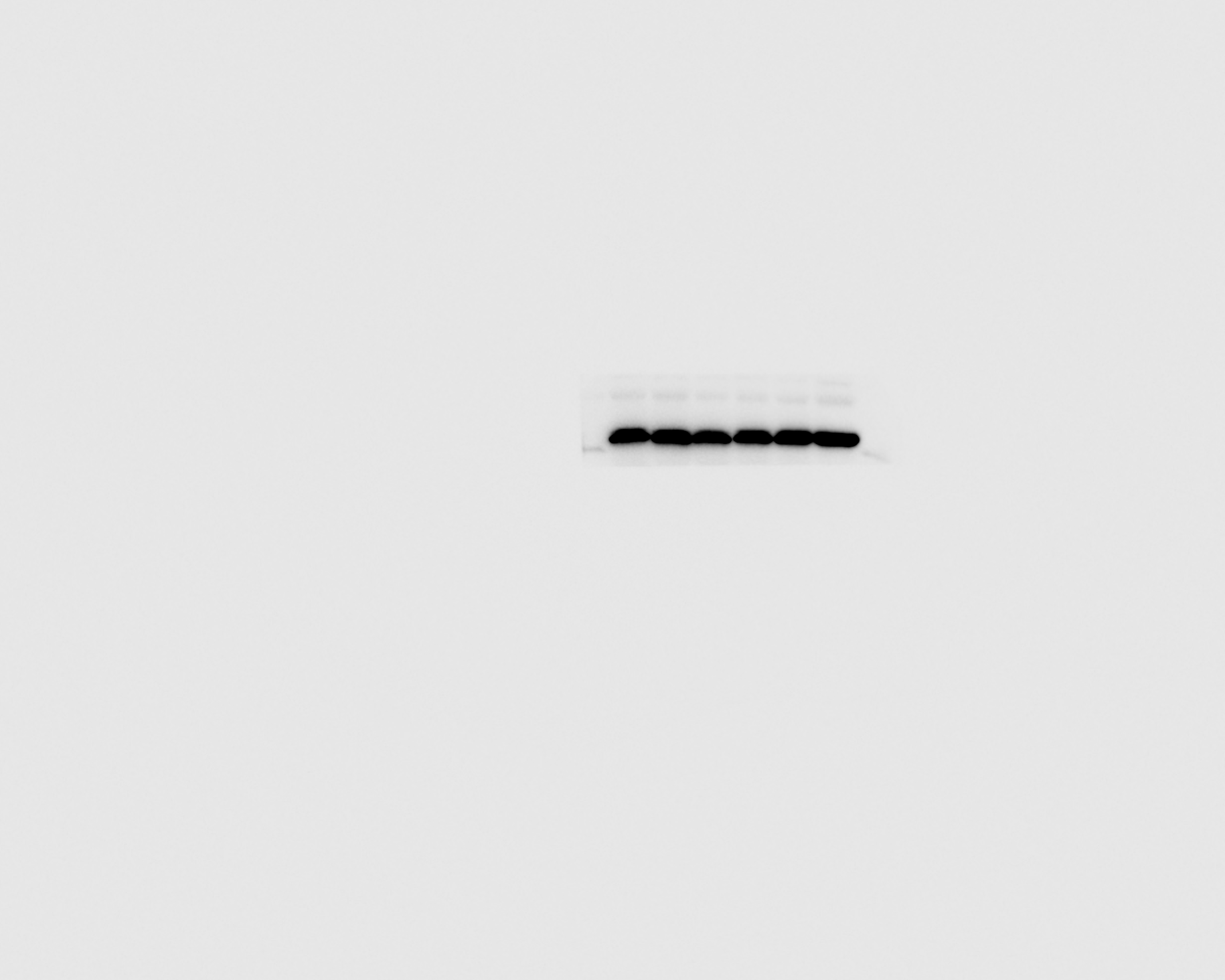

Supplement: Supplemental Information 2 [file peerj-11-15180-s002.zip › Figure7 full-length uncropped blots and replicates/c-jun-D.tif]

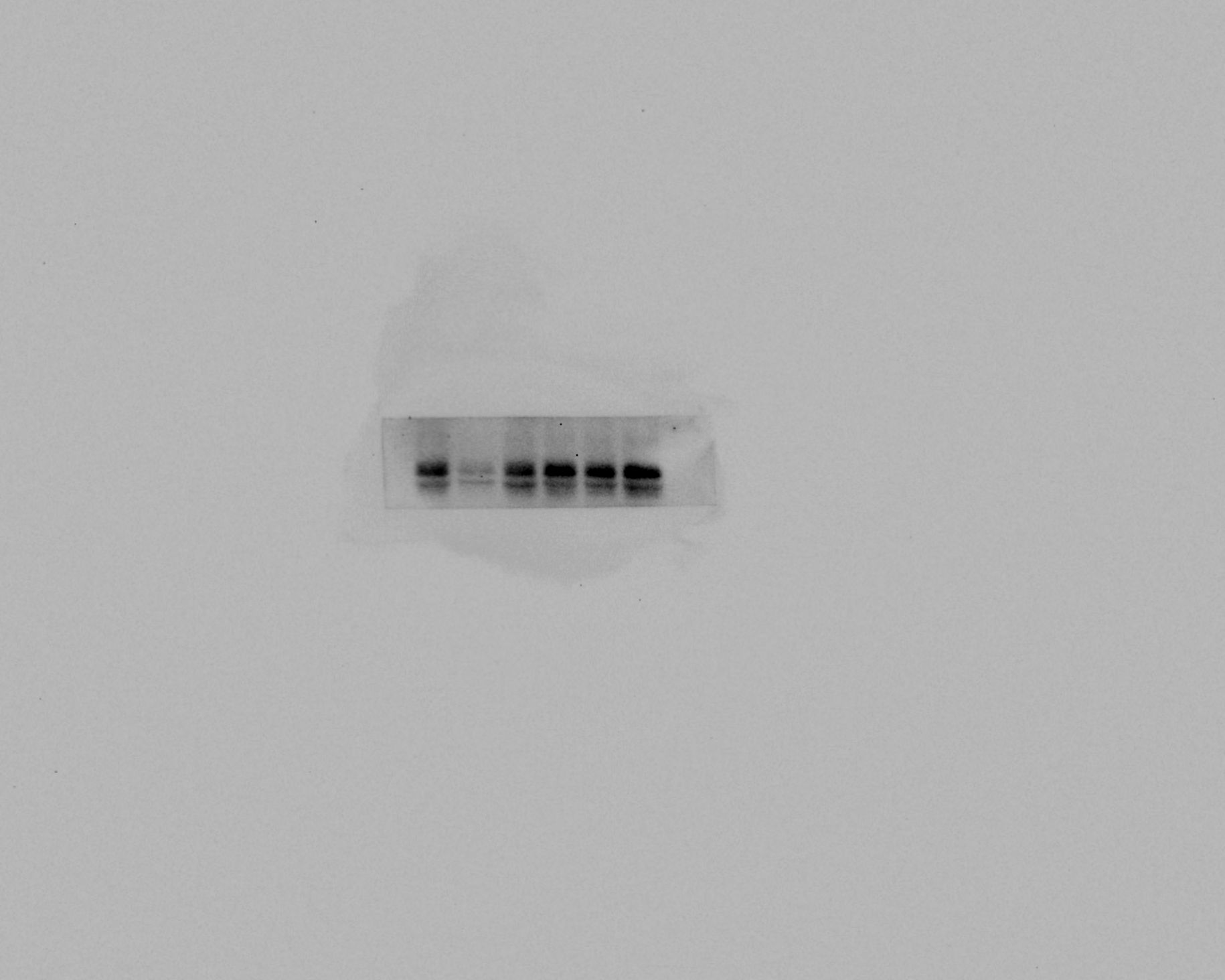

Supplement: Supplemental Information 2 [file peerj-11-15180-s002.zip › Figure7 full-length uncropped blots and replicates/KLF4-A.tif]

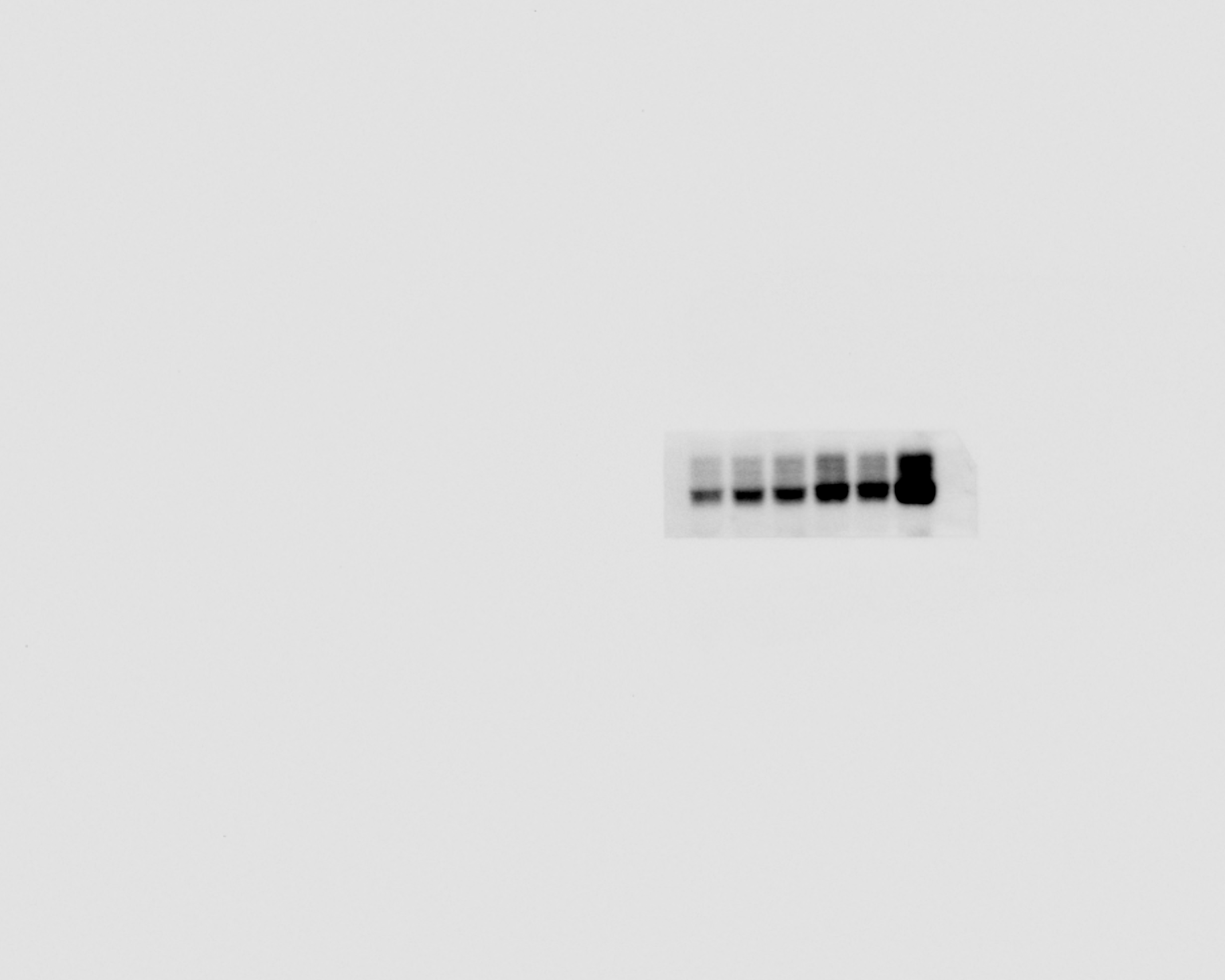

Supplement: Supplemental Information 2 [file peerj-11-15180-s002.zip › Figure7 full-length uncropped blots and replicates/KLF4-C.tif]

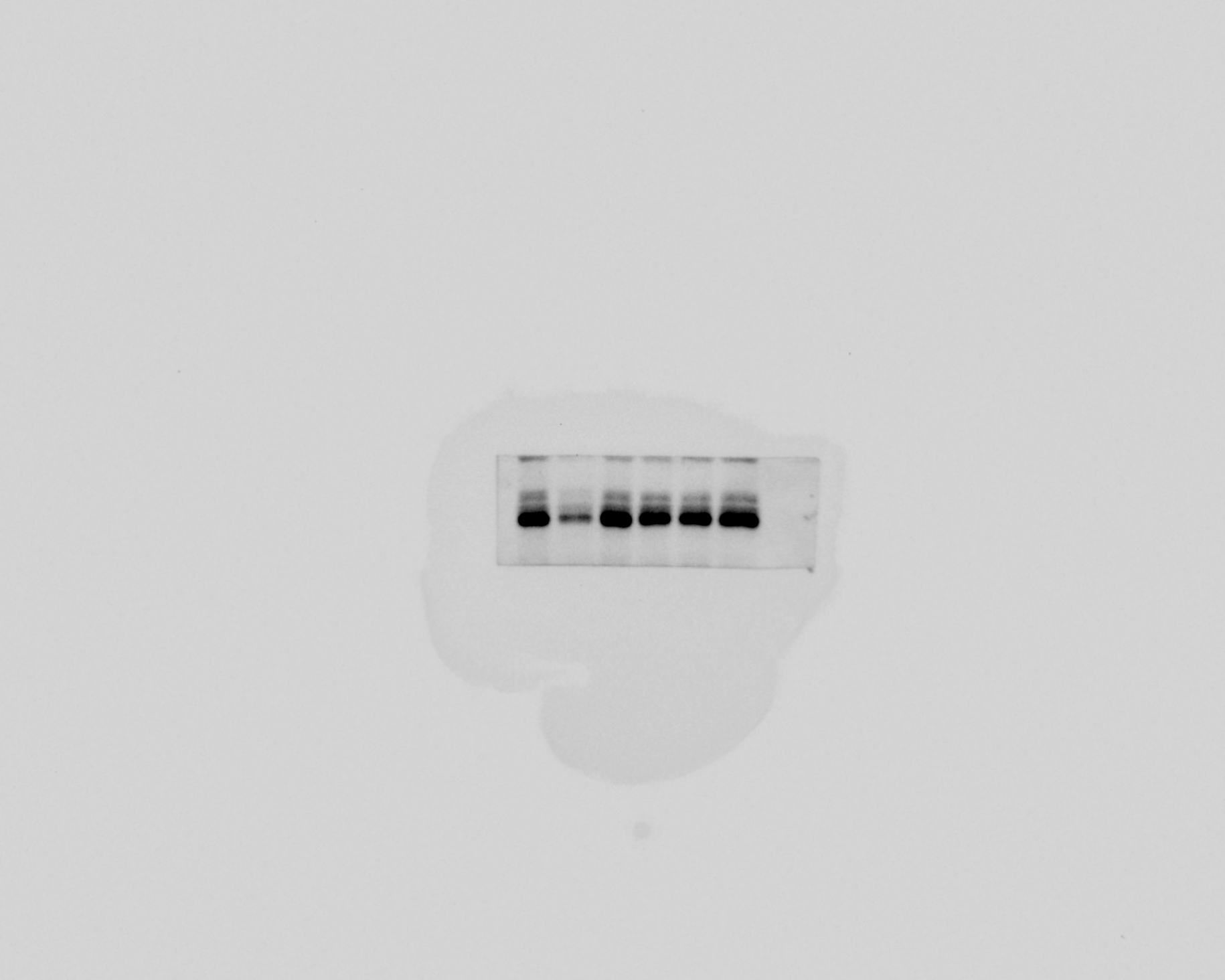

Supplement: Supplemental Information 2 [file peerj-11-15180-s002.zip › Figure7 full-length uncropped blots and replicates/KLF4-B.tif]

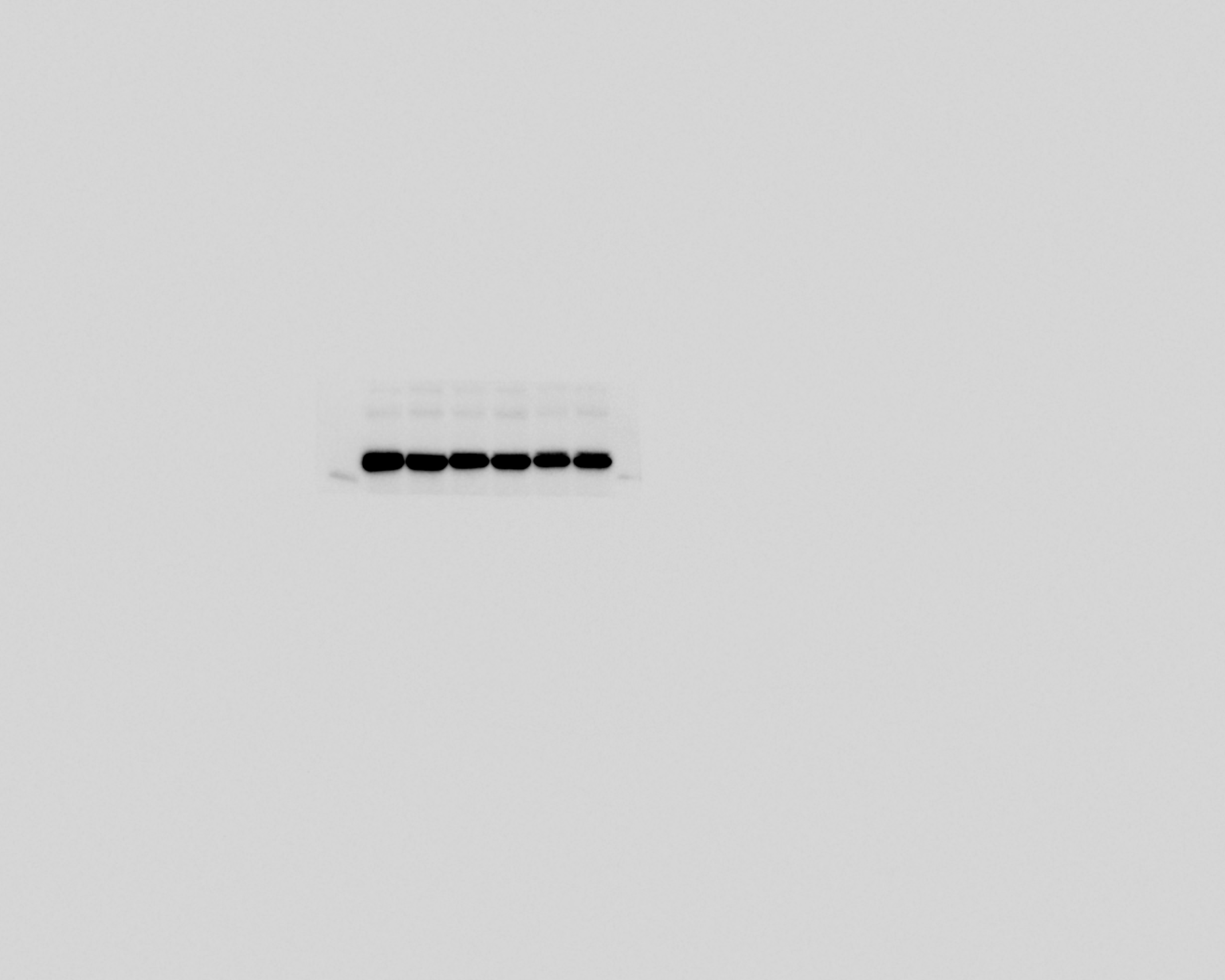

Supplement: Supplemental Information 2 [file peerj-11-15180-s002.zip › Figure7 full-length uncropped blots and replicates/c-jun-B.tif]

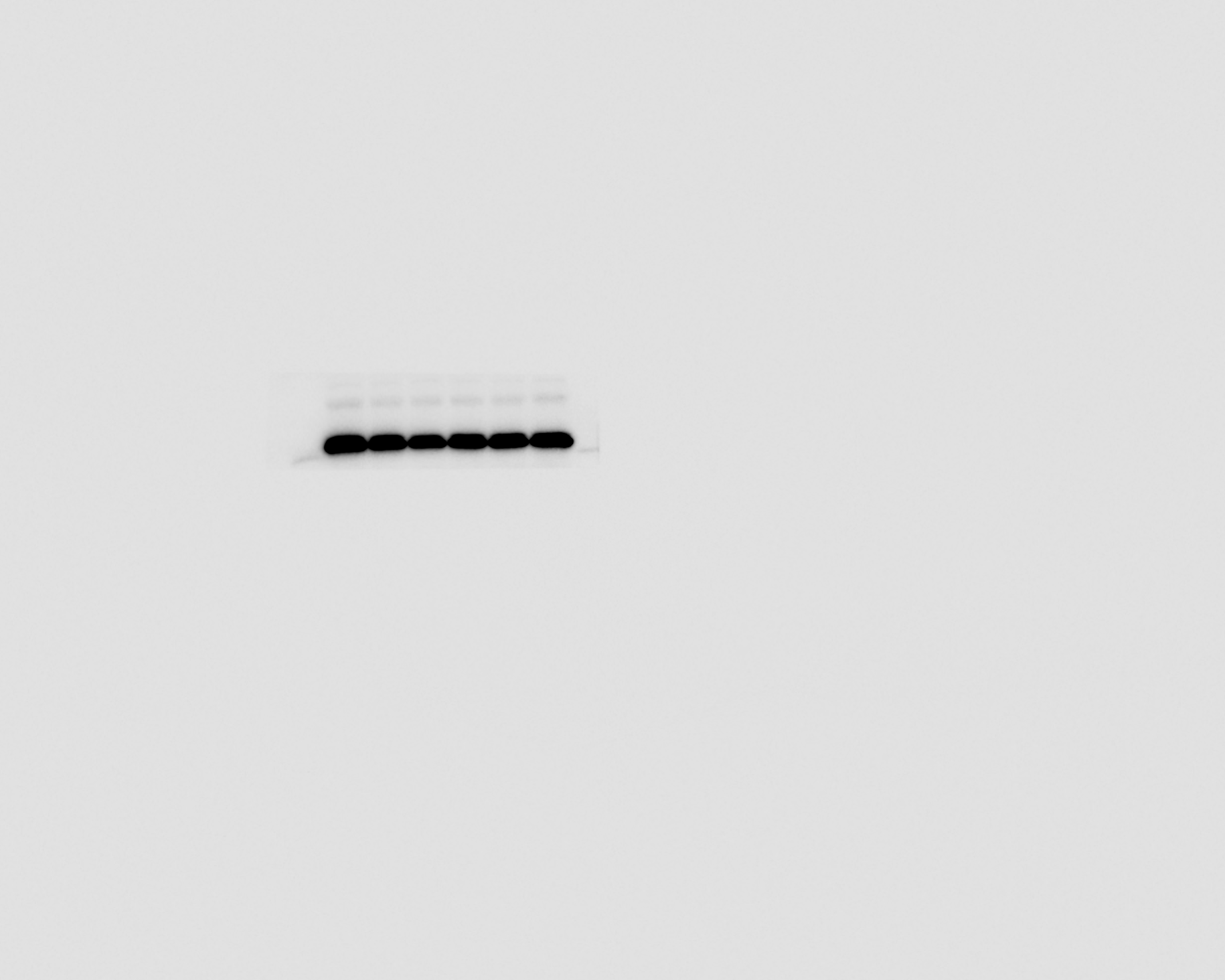

Supplement: Supplemental Information 2 [file peerj-11-15180-s002.zip › Figure7 full-length uncropped blots and replicates/c-jun-C.tif]

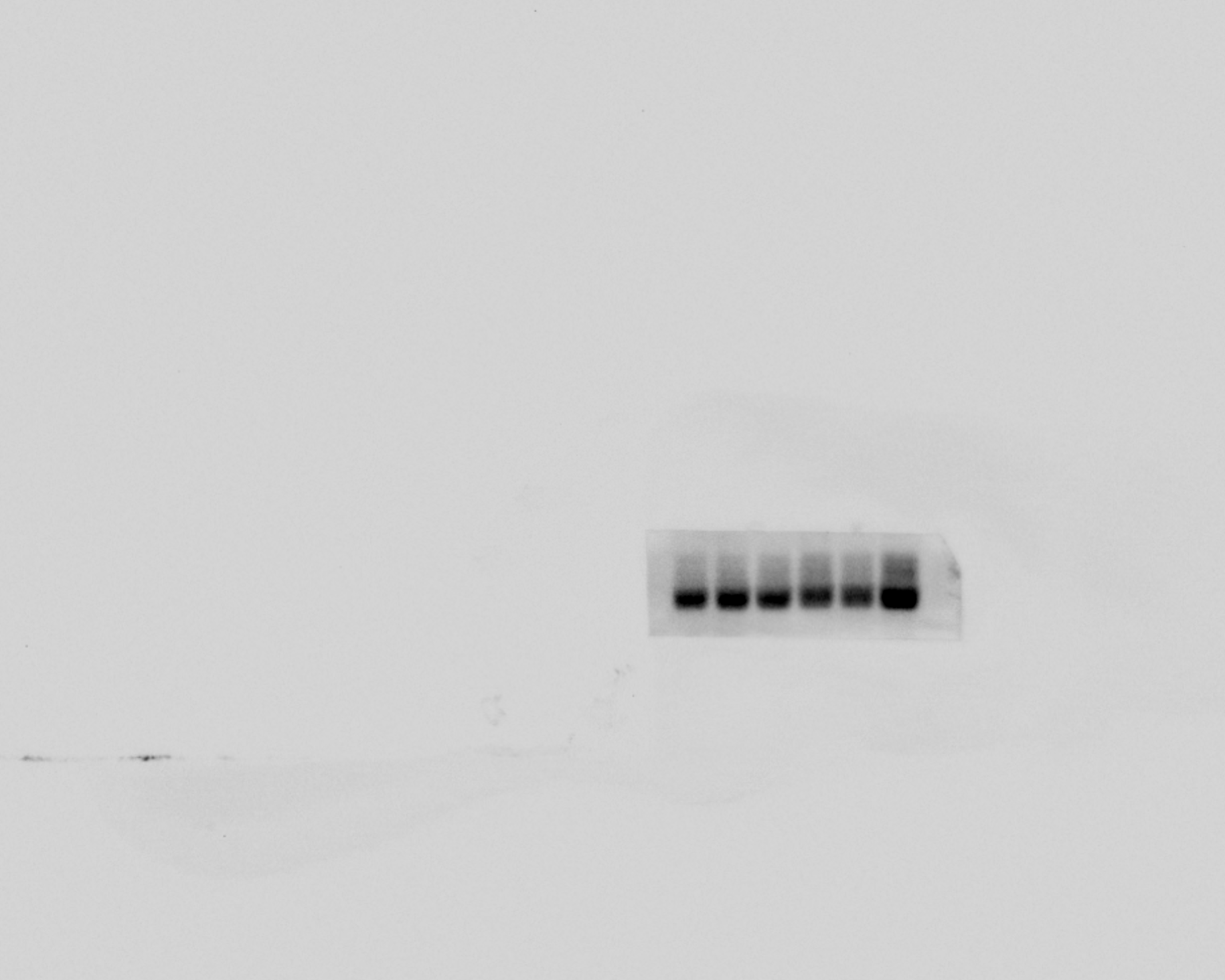

Supplement: Supplemental Information 2 [file peerj-11-15180-s002.zip › Figure7 full-length uncropped blots and replicates/c-jun-A.tif]

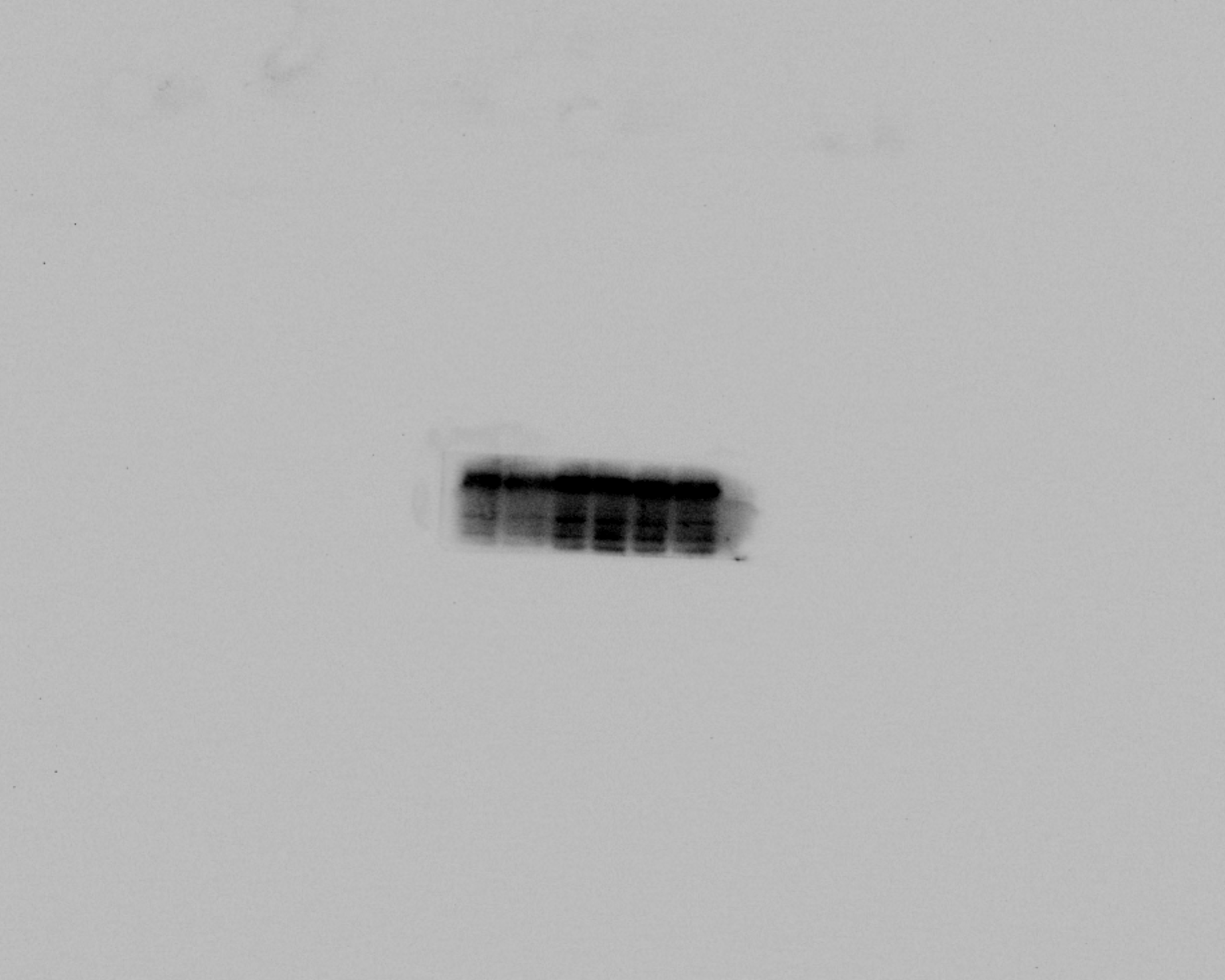

Supplement: Supplemental Information 2 [file peerj-11-15180-s002.zip › Figure7 full-length uncropped blots and replicates/KLF4-D.tif]

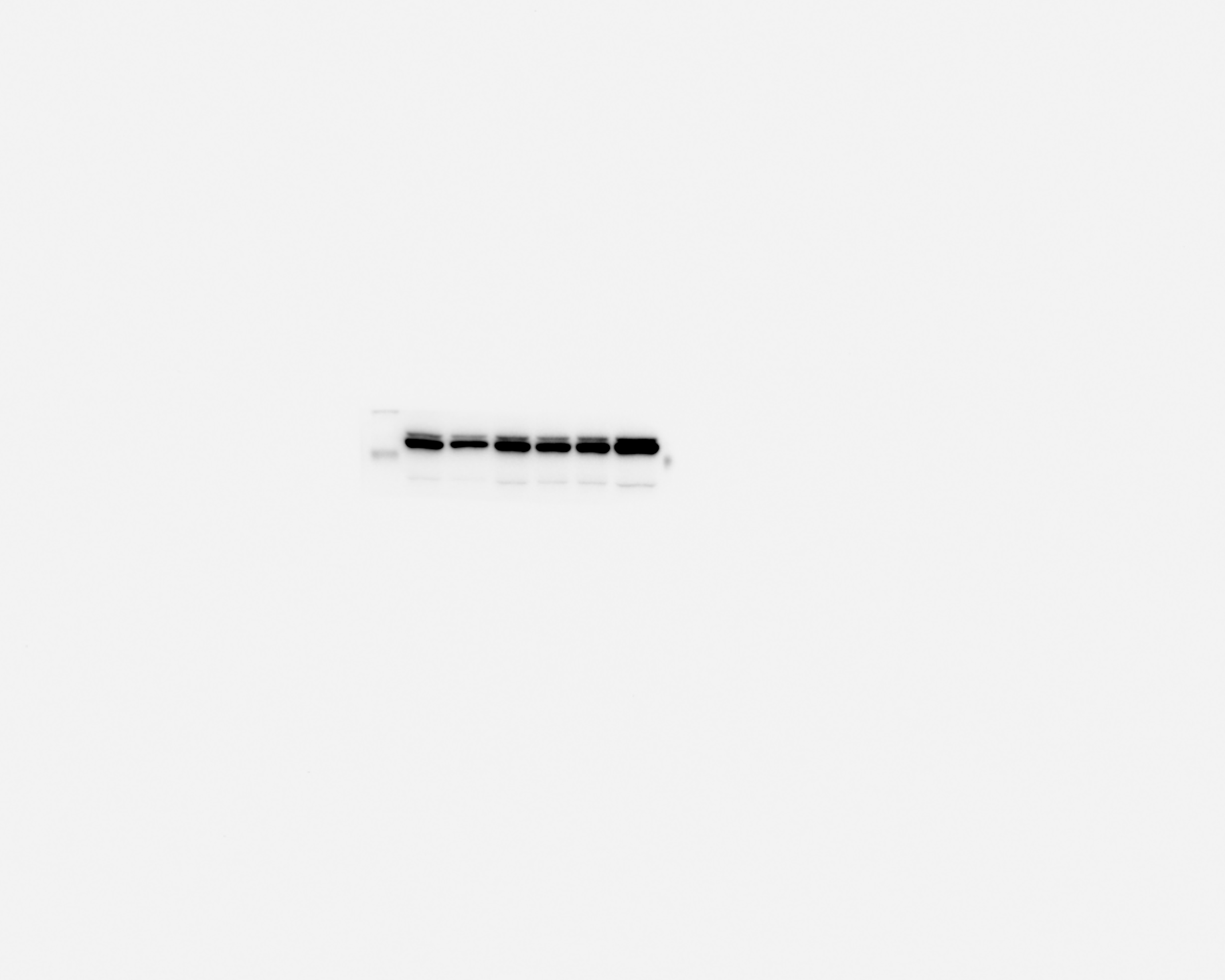

Supplement: Supplemental Information 2 [file peerj-11-15180-s002.zip › Figure7 full-length uncropped blots and replicates/c-fos-A.tif]

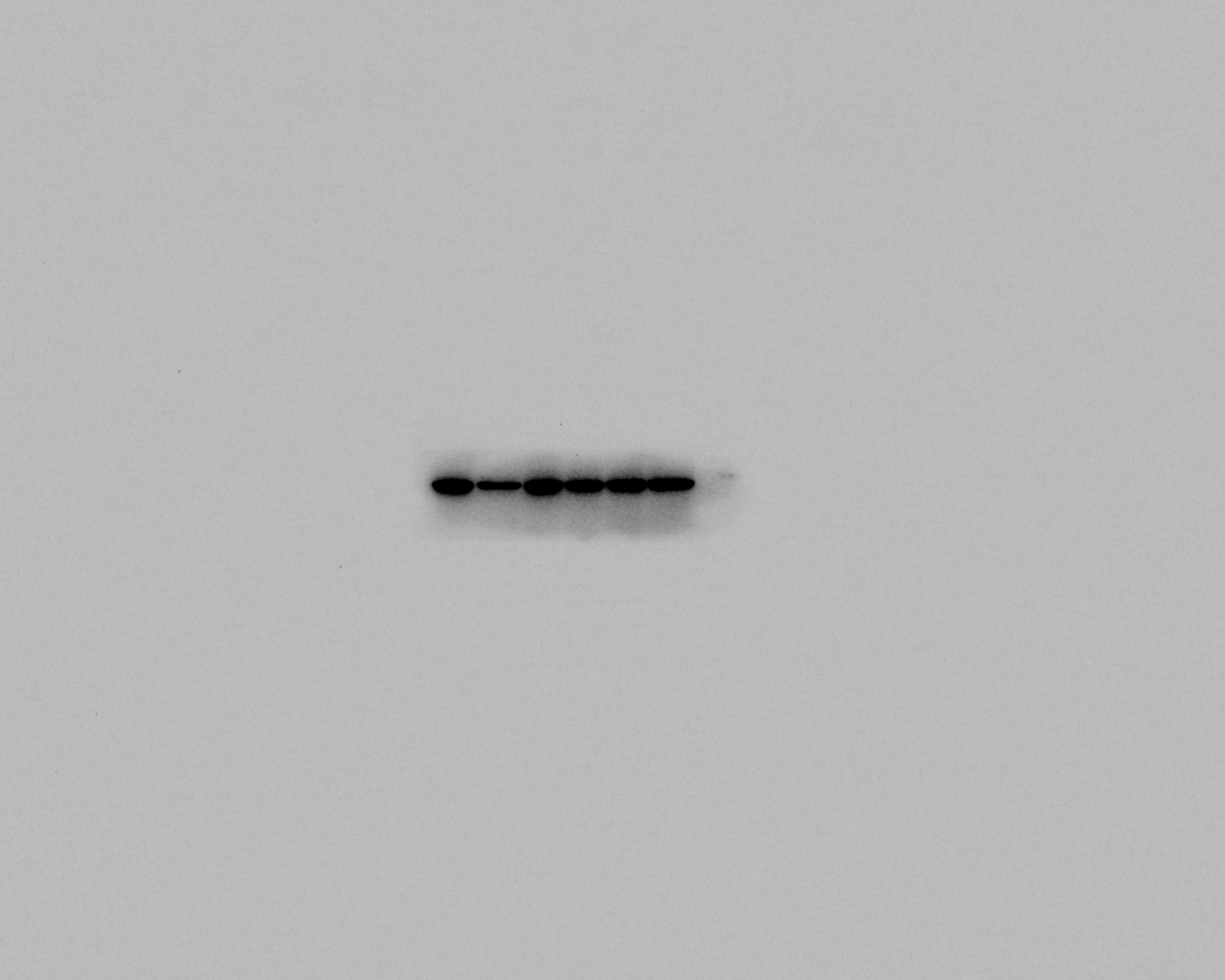

Supplement: Supplemental Information 2 [file peerj-11-15180-s002.zip › Figure7 full-length uncropped blots and replicates/c-fos-B.tif]

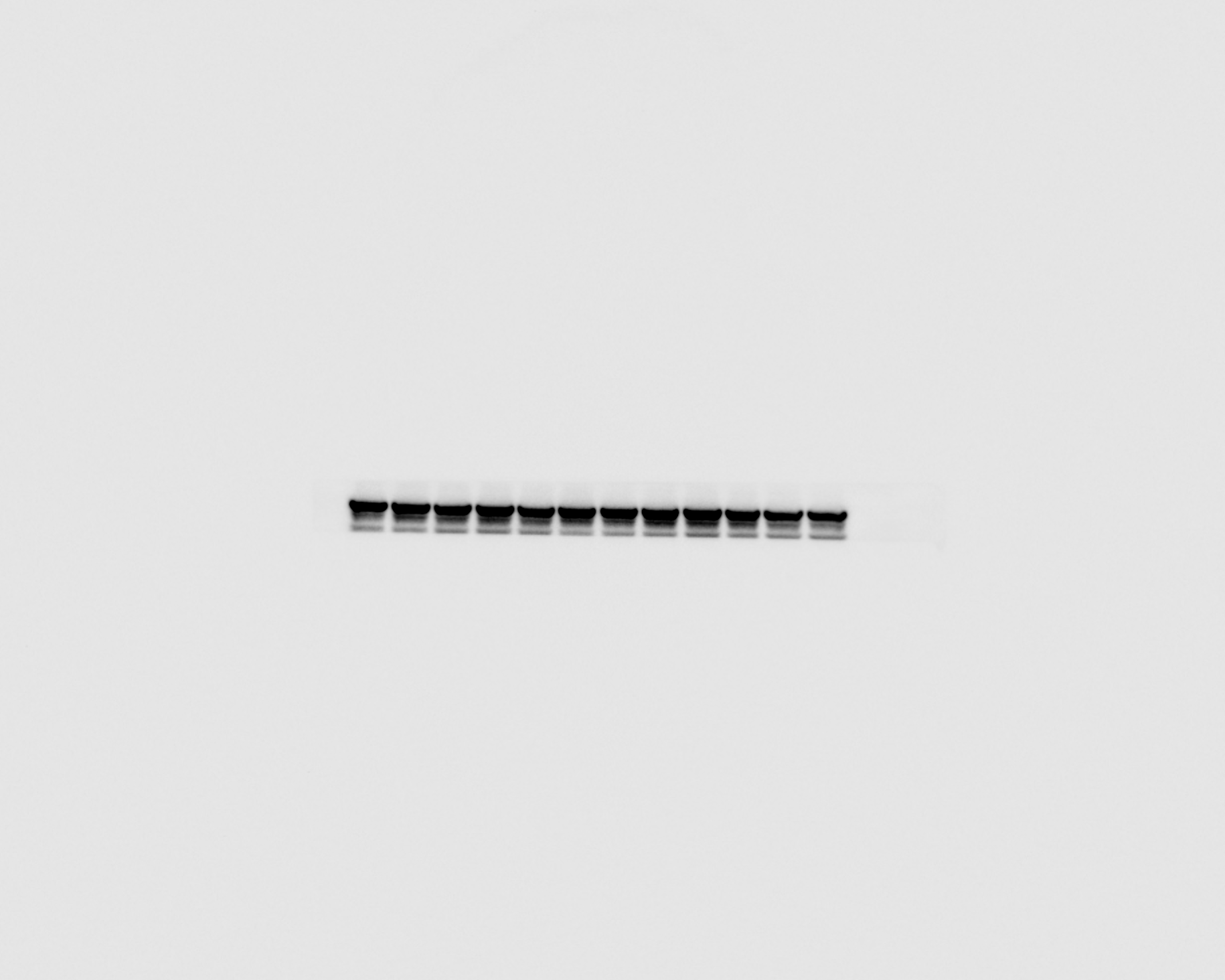

Supplement: Supplemental Information 2 [file peerj-11-15180-s002.zip › Figure7 full-length uncropped blots and replicates/Actin-EF.tif]

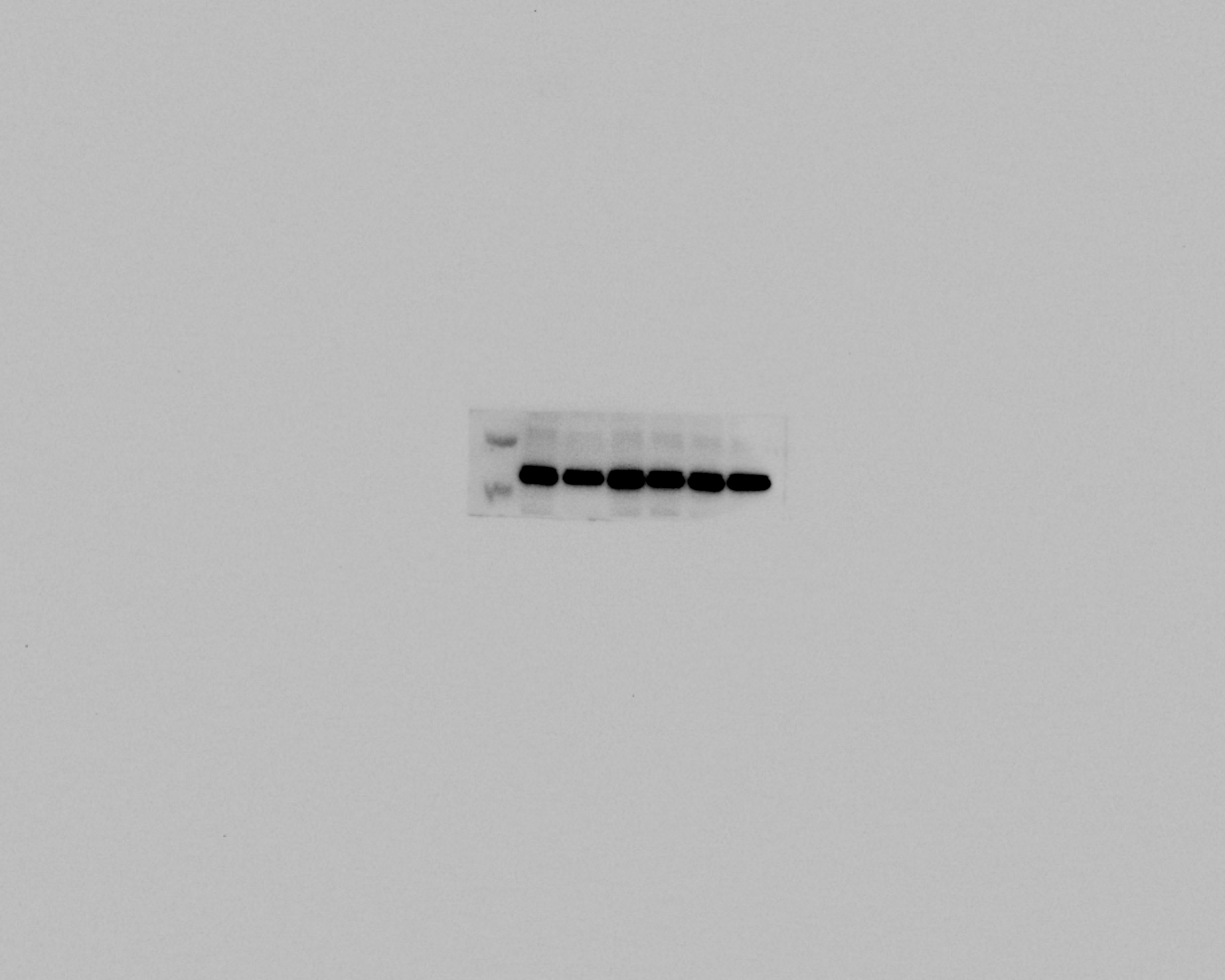

Supplement: Supplemental Information 2 [file peerj-11-15180-s002.zip › Figure7 full-length uncropped blots and replicates/c-fos-C.tif]

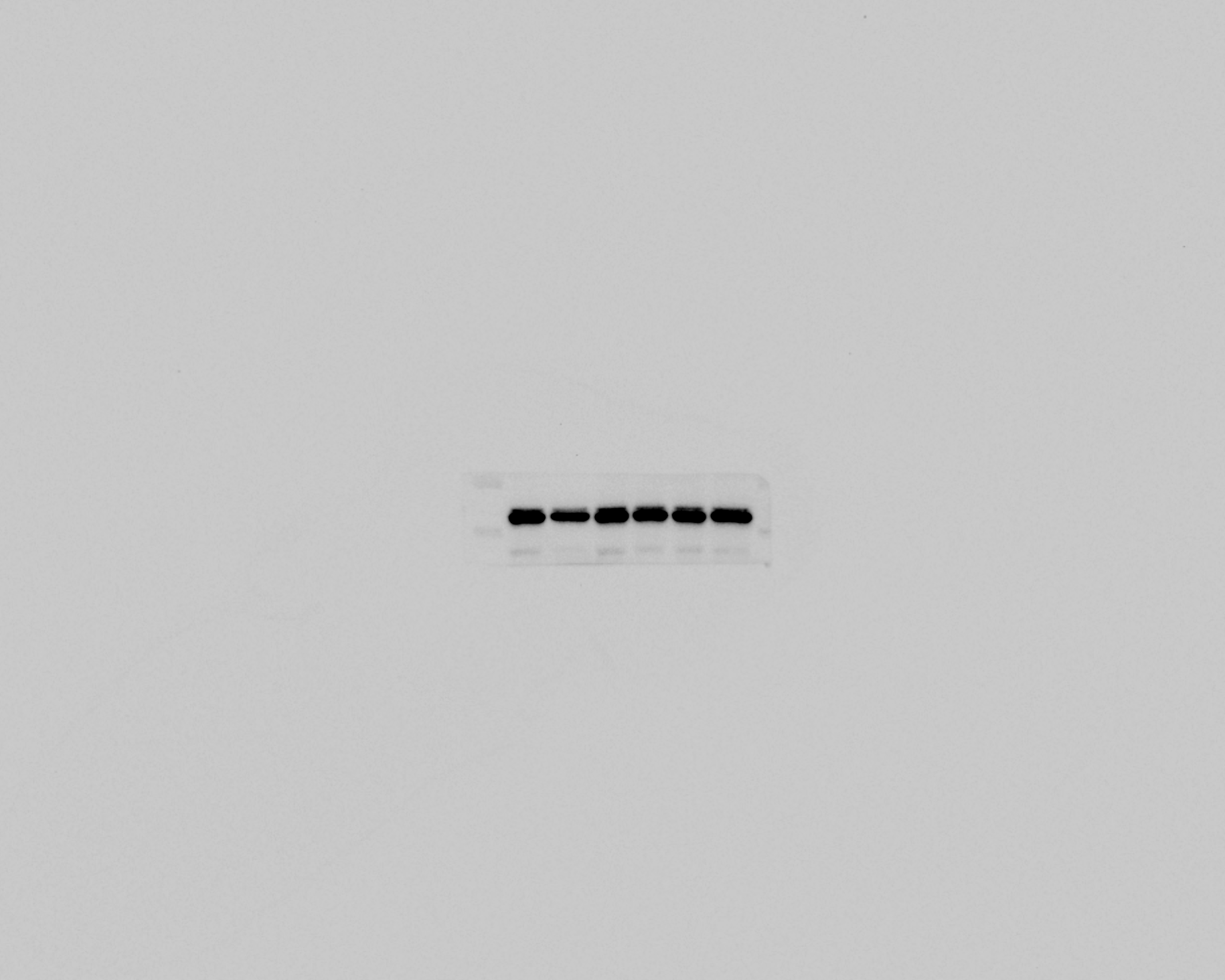

Supplement: Supplemental Information 2 [file peerj-11-15180-s002.zip › Figure7 full-length uncropped blots and replicates/c-fos-D.tif]

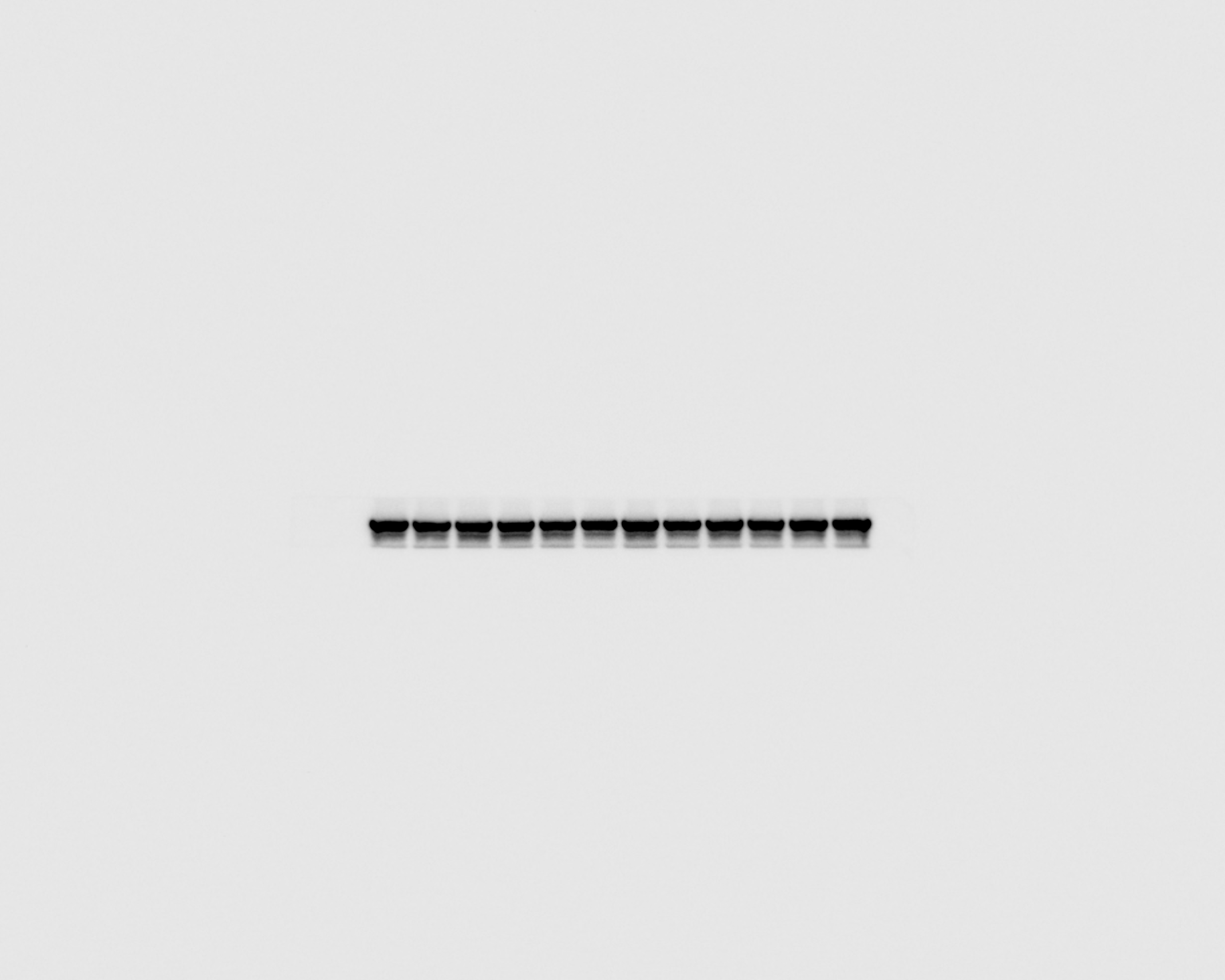

Supplement: Supplemental Information 2 [file peerj-11-15180-s002.zip › Figure7 full-length uncropped blots and replicates/Actin-GH.tif]

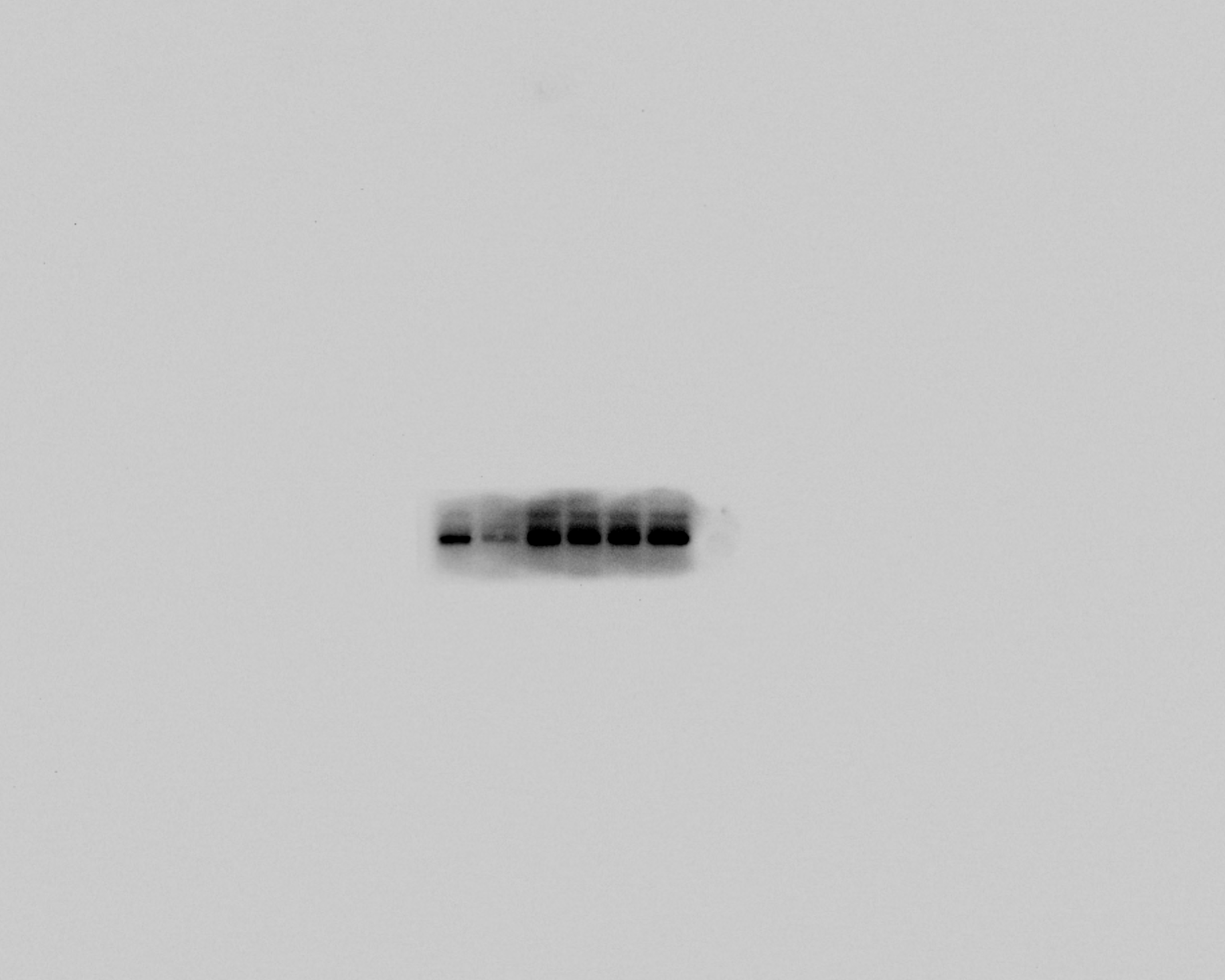

Supplement: Supplemental Information 2 [file peerj-11-15180-s002.zip › Figure7 full-length uncropped blots and replicates/VEGF-D.tif]

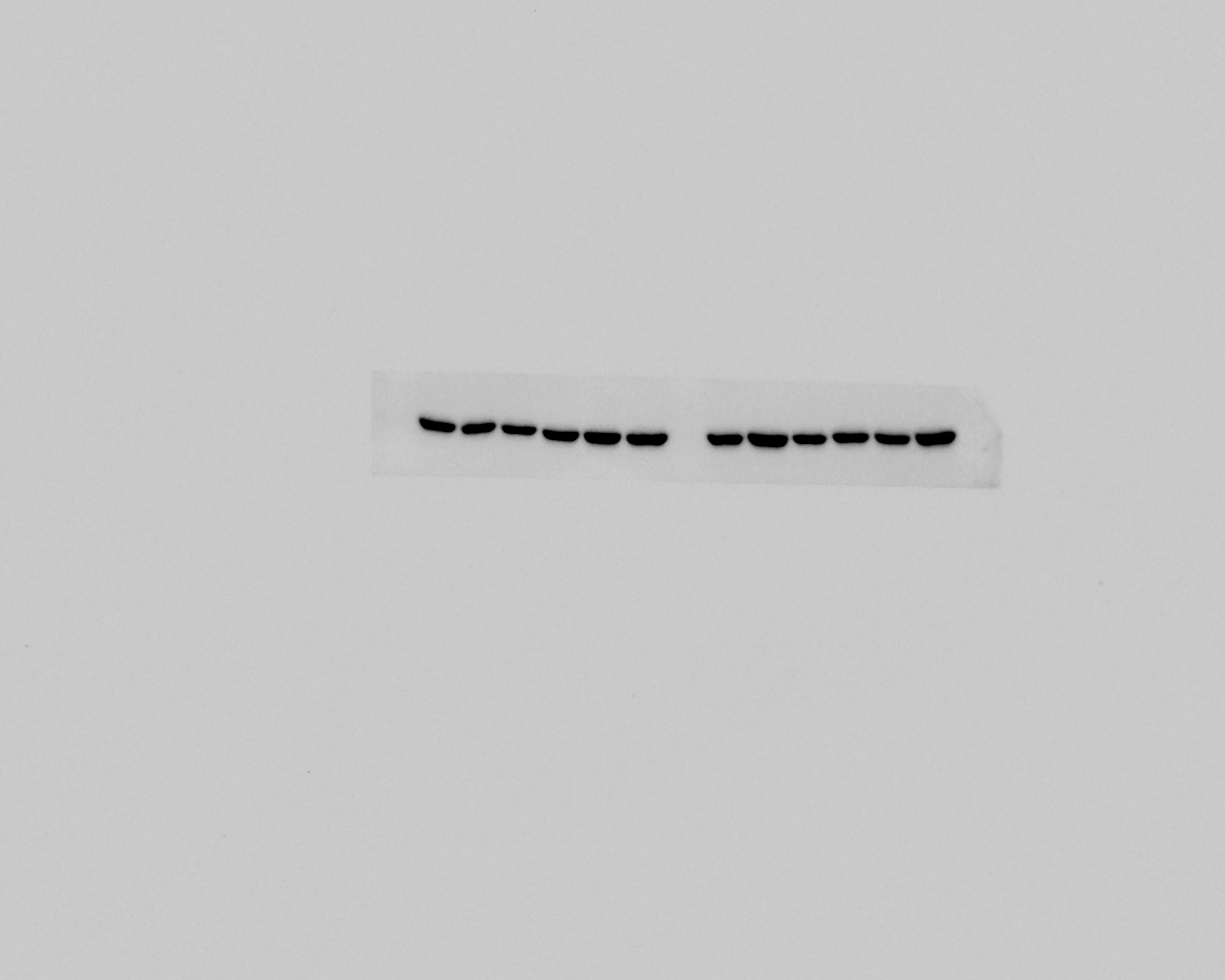

Supplement: Supplemental Information 2 [file peerj-11-15180-s002.zip › Figure7 full-length uncropped blots and replicates/Actin-CD.tif]

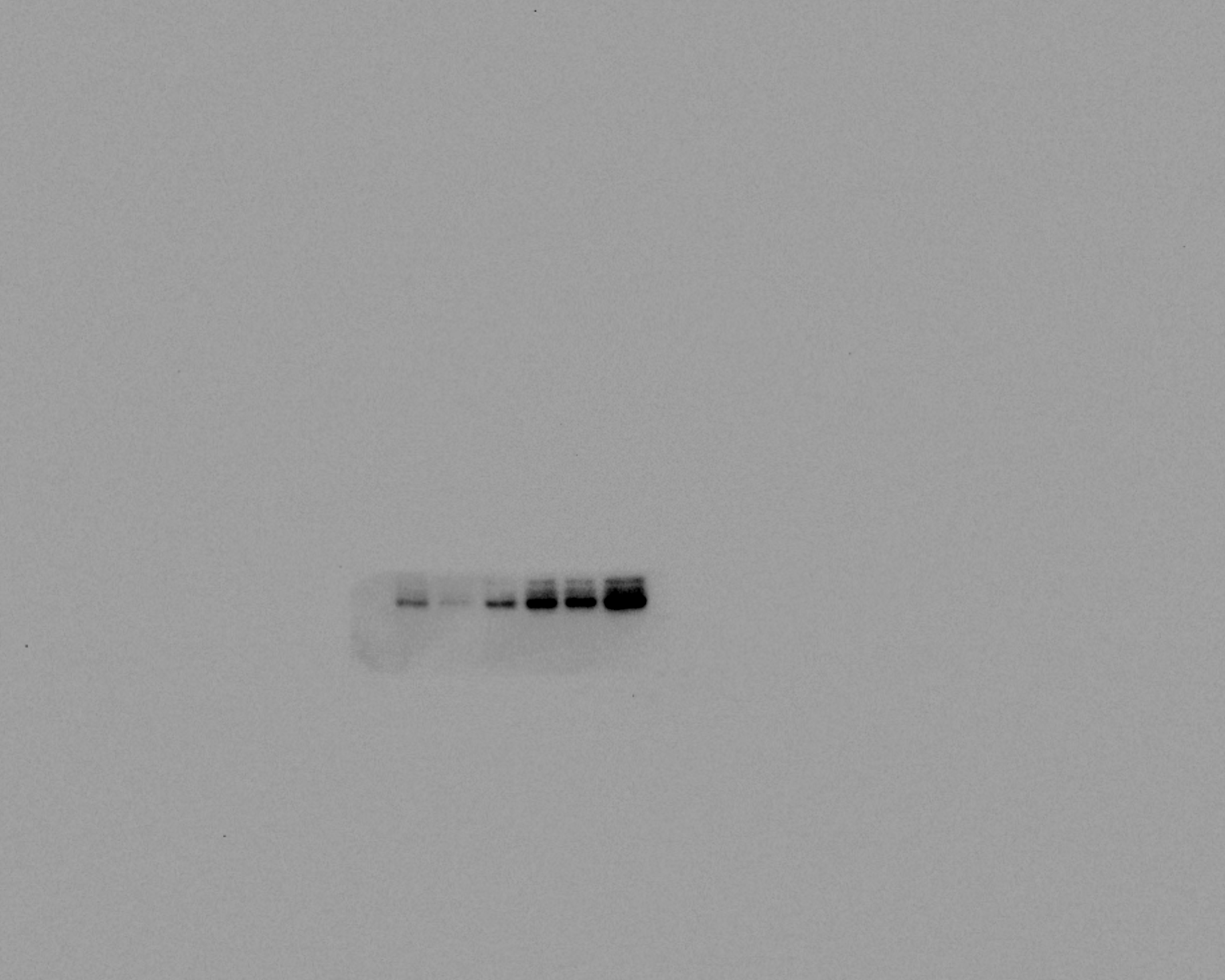

Supplement: Supplemental Information 2 [file peerj-11-15180-s002.zip › Figure7 full-length uncropped blots and replicates/VEGF-A.tif]

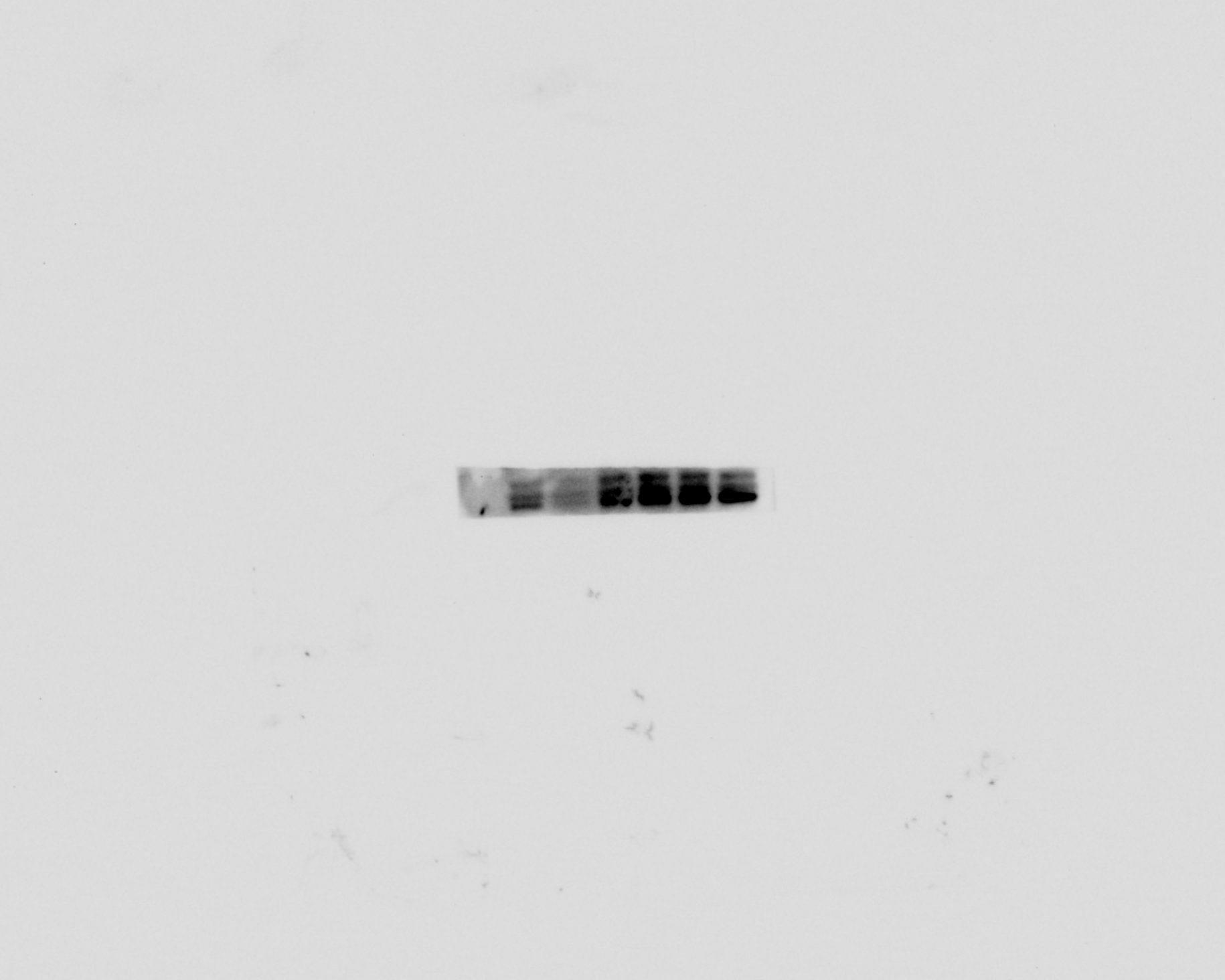

Supplement: Supplemental Information 2 [file peerj-11-15180-s002.zip › Figure7 full-length uncropped blots and replicates/VEGF-B.tif]

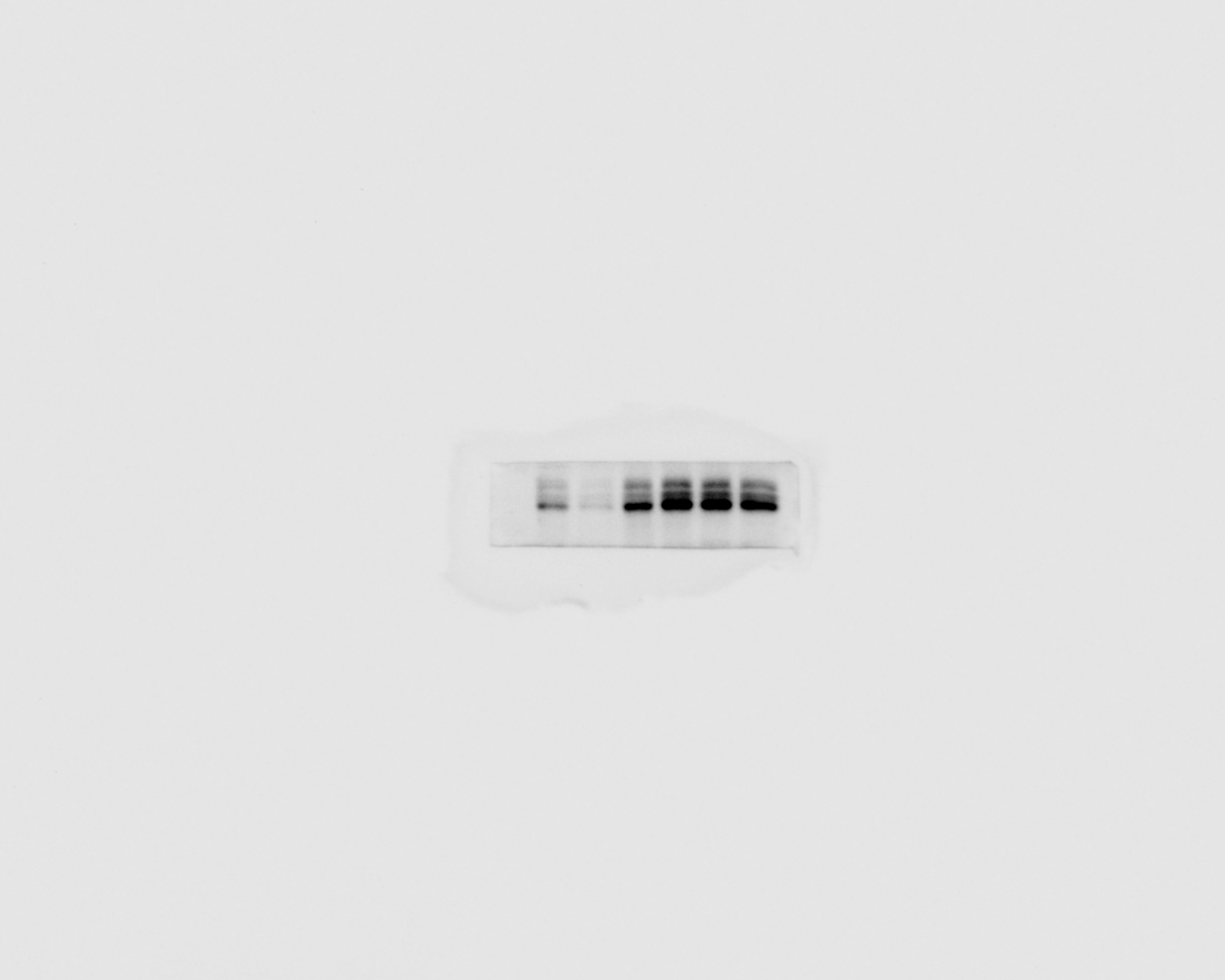

Supplement: Supplemental Information 2 [file peerj-11-15180-s002.zip › Figure7 full-length uncropped blots and replicates/VEGF-C.tif]

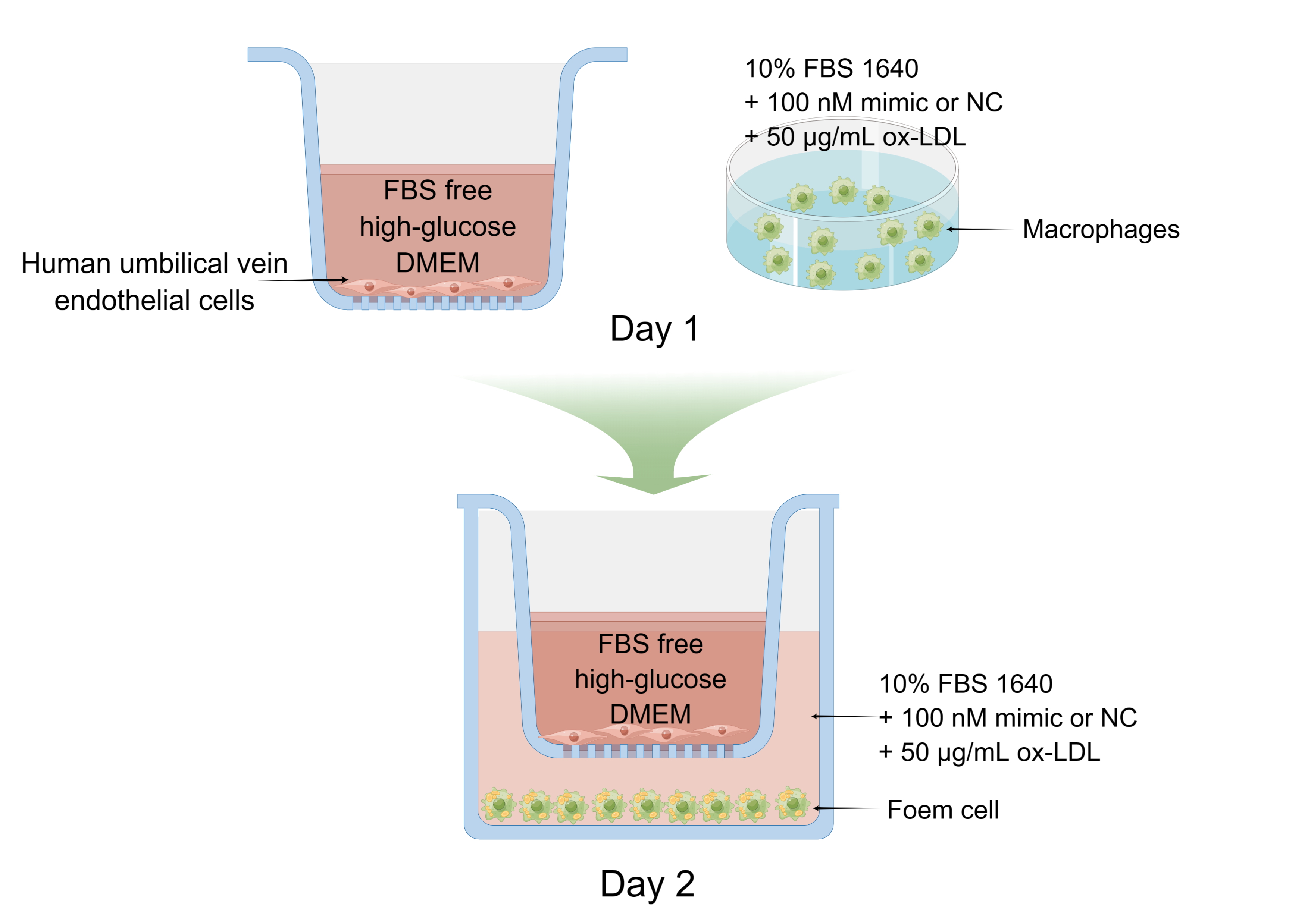

Supplement: Supplemental Information 3 [file peerj-11-15180-s003.png]
